# Supplementary material for: Regulation of mycobacterial infection by macrophage Gch1 and tetrahydrobiopterin
Source: Nat Commun. 2018 Dec 20;9:5409. doi: 10.1038/s41467-018-07714-9 (PMC6302098; doi:10.1038/s41467-018-07714-9)
Supplement: Supplementary file 1 — Supplementary Information [file 41467_2018_7714_MOESM1_ESM.pdf]

Regulation of Mycobacterial Infection by Macrophage *Gch1* and Tetrahydrobiopterin

McNeill *et al.*

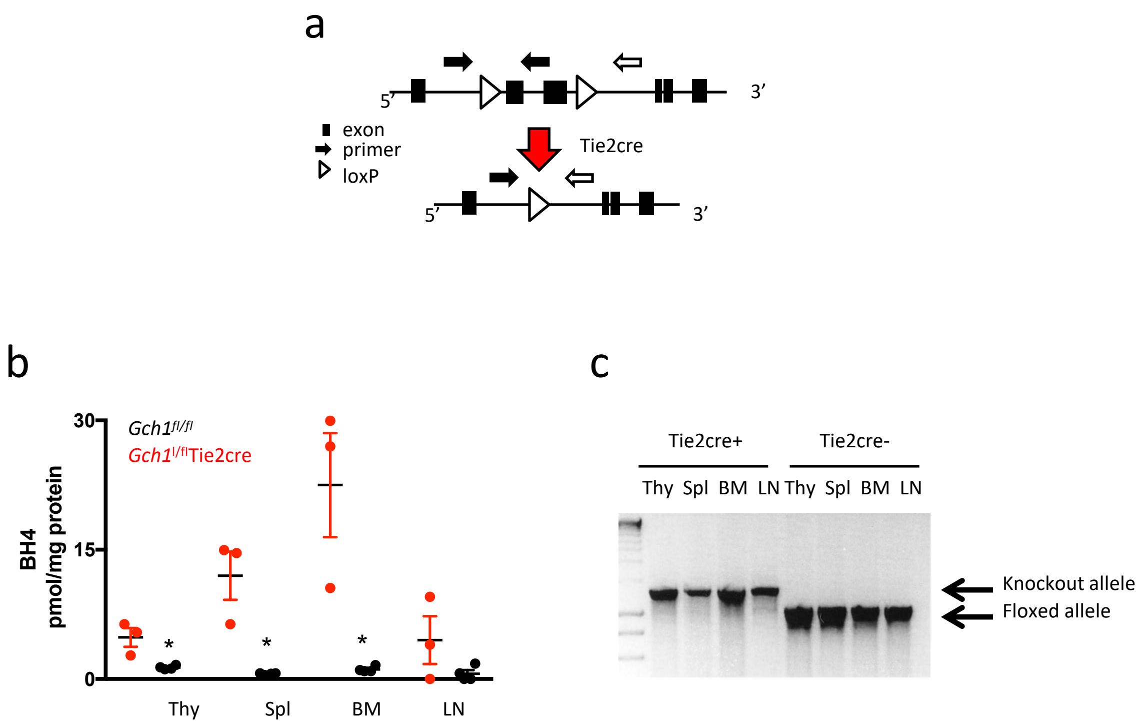

Supplementary Figure 1: *Gch1*$^{fl/fl}$Tie2cre mice exhibit a widespread knockout of *Gch1* throughout the hematopoietic system. (a) Schematic showing the targeting of the mouse *Gch1* locus indicating the position of the loxP sites flanking exons 2 and 3, which encode the active site of the GTPCH enzyme. Arrows indicate the position of primers that produce the product from the floxed allele (solid arrows) and excised allele (solid and open arrows). **(b)** Cell suspensions underwent biopterin analysis by HPLC to quantify BH4 content in leukocyte isolations from thymus (Thy), spleen (Spl), bone marrow (BM) and lymph node (LN) (E). **(c)** Genomic PCR confirmed floxed allele excision in leukocyte populations. (individual points shown, error bars +/- SEM).

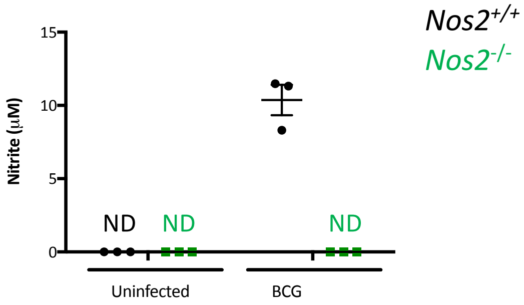

Supplementary Figure 2: The origin and specificity of the nitrite signal detected in cell culture supernatants was confirmed by infection of BMDM from iNOS$^{-/-}$ mice. BMDM were prepared from *Nos2*$^{+/+}$and age-matched iNOS$^{-/-}$ animals. Macrophages were infected at a MOI of 1:1 with BCG in the presence of 10ng/ml IFNγ, or left uninfected. Cell culture supernatants were collected after 24hours and nitrite accumulation quantified by the Griess assay (n=3 per group, error bars +/- SEM, ND: not detected).

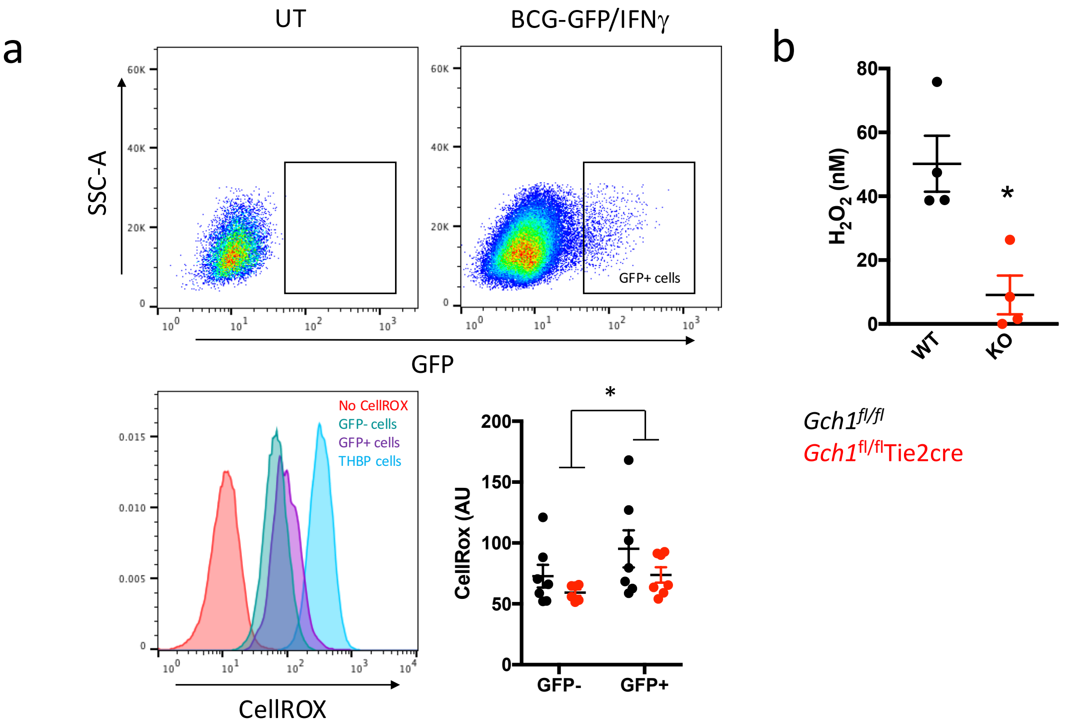

Supplementary Figure 3: Redox changes in BH4 deficient macrophages are complex.
(a) *Gch1*$^{fl/fl}$Tie2cre and *Gch1*$^{fl/fl}$ macrophages were stimulated IFNγ (10ng/ml) and BCG (MOI 5:1) for 2 hours. CellROX cell permeable ROS indicator dye was added to the media at 500nM for an additional 30min. At the end of the assay the cells were washed twice, harvested and analysed by flow cytometry within 120mins. GFP+ cells were gated by comparison to non-infected controls and the Cell ROX signal quantified in the GFP+ and GFP- populations. Tertbutyl hydroperoxide (THBP) was included as a positive control to induce oxidative stress. Data was analysed by 2-way ANOVA * $p<0.05$. No significant effect of genotype was detected by post-testing (n=6-7/genotype). **(b)** H_2O_2 accumulation in the cell culture media over 24hrs of cell stimulation with BCG/IFNγ from *Gchf1lf/fl* and *Gch1fl/flTie2cre* bone marrow derived macrophages was measured using a hydrogen peroxide electrode (n=4/genotype) * $p<0.05$ two-tailed T-test.

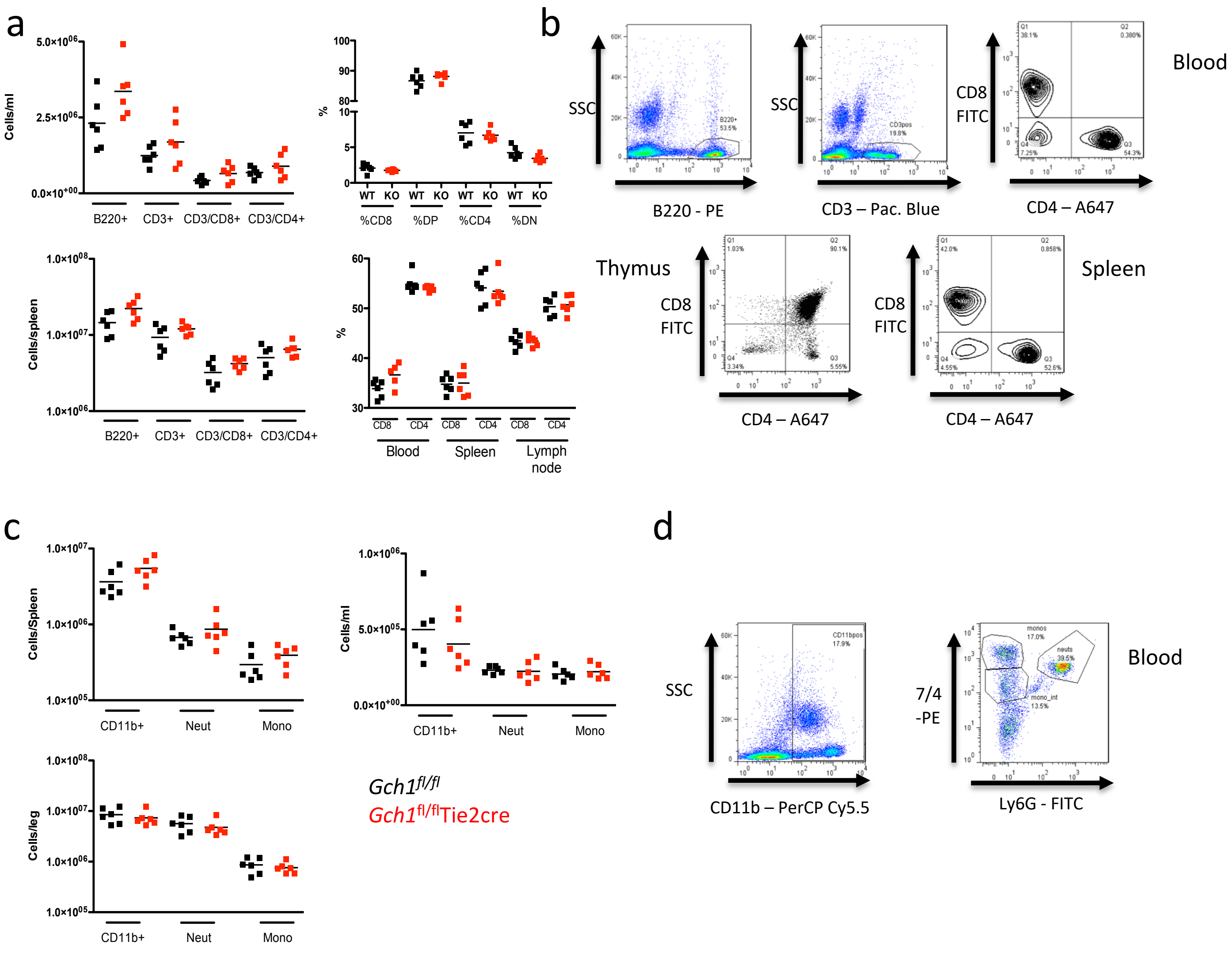

Supplementary Figure 4: *Gch1*$^{fl/fl}$Tie2cre mice exhibit a widespread knockout of *Gch1* throughout the hematopoietic system, but with no significant effect on homeostatic leukocyte cells numbers. Blood, bone marrow, thymus, spleen and lymph node were isolated and single cell suspensions produced. Quantification of peripheral lymphocyte numbers in blood and spleen, thymic lymphocyte composition and peripheral CD4/CD8 ratios. (No significant difference, t-test, individual points shown) **(a)**. Representative flow cytometry data for lymphoycte analysis **(b)**. Quantification of total myeloid cells number (CD11b+), neutrophils (7/4^{HI}/Ly-6G^{+}) and monocytes (7/4^{HI} Ly-6G^{-}) **(c)**. Representative flow cytometry data for myeloid cell analysis **(d)**.(t-test, n=6 per group).

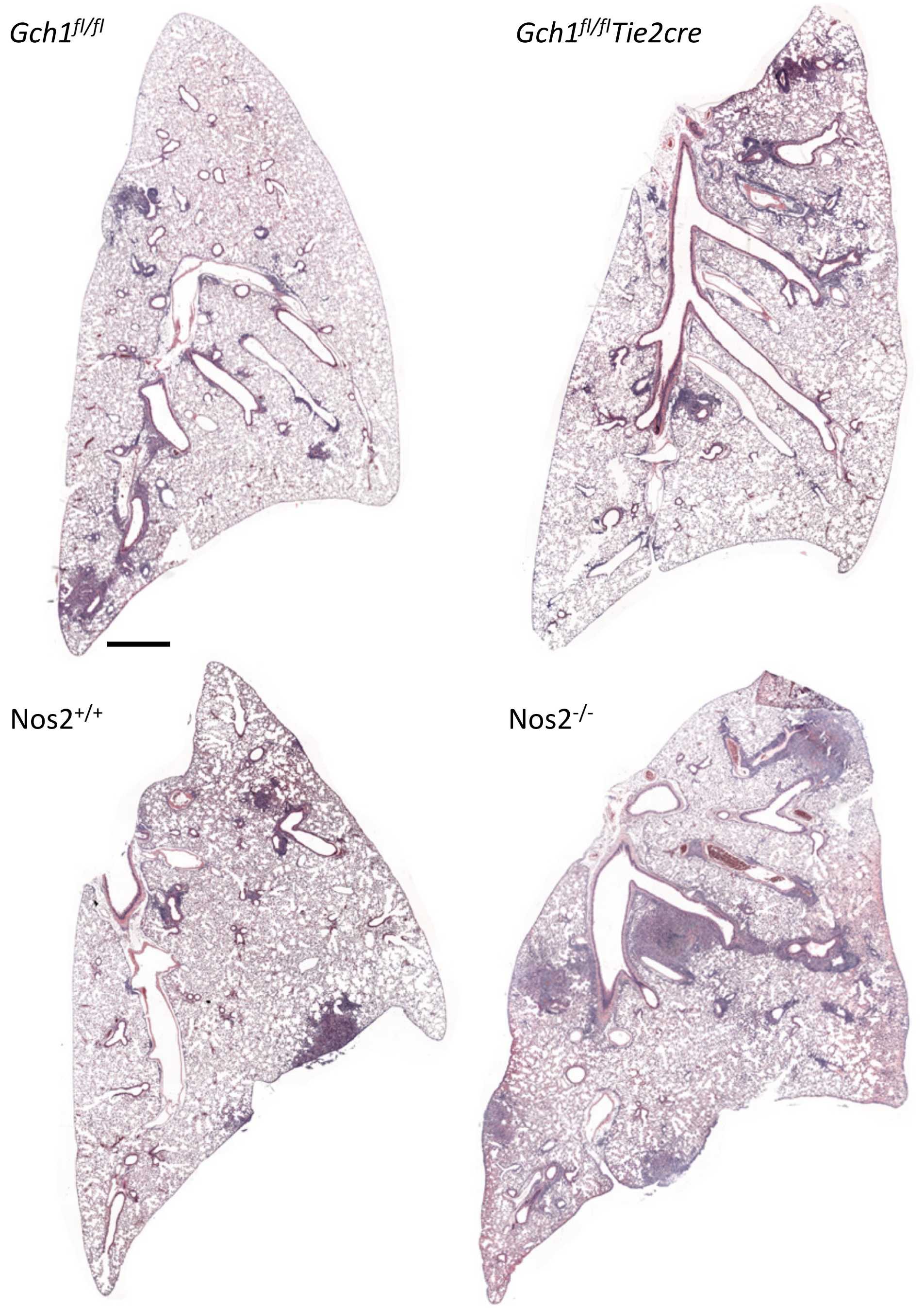

Supplementary Figure 5: Lung gross morphology. Mice were infected with 150 CFU *M.tb* (Erdman strain) by aerosol inhalation. 23 days following infection the lungs were removed for analysis. Representative organs were imaged from each genotype prior to n=2 tissues per genotype being embedded for histochemical analysis. Sections were cut from all tissues and and stained with H&E. Tiled images of the whole lung were produced. Scale bar 1mm.

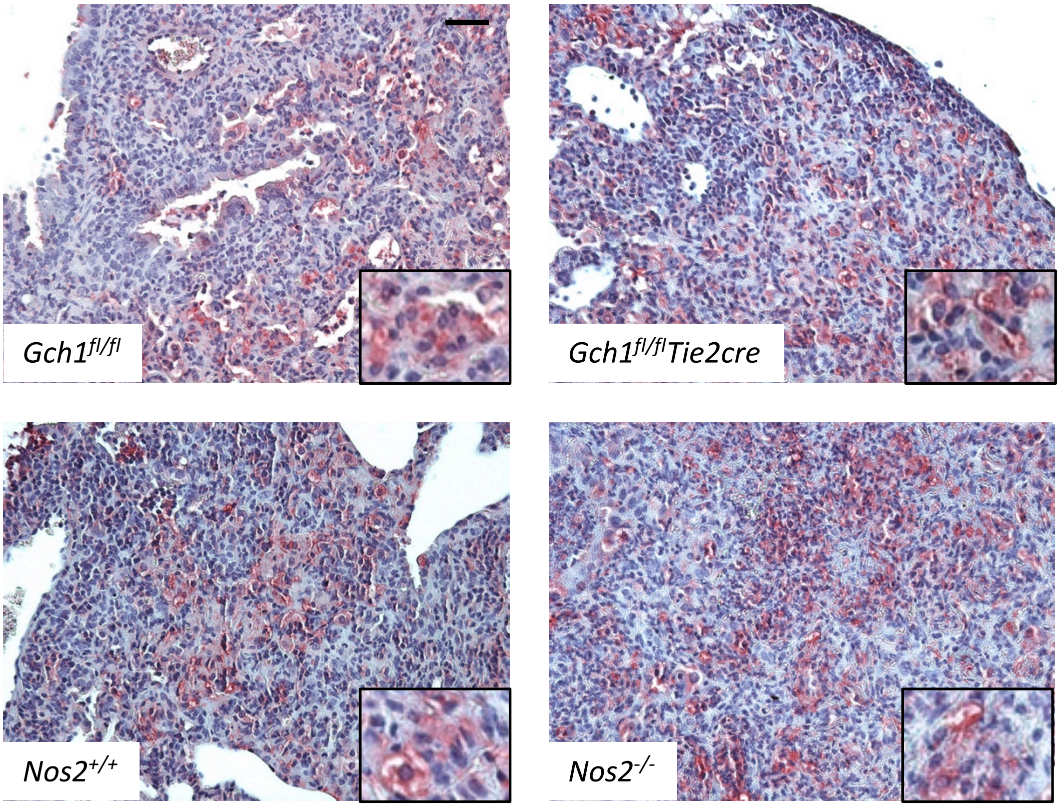

Supplementary Figure 6: Macrophage localisation to granuloma. Mice were infected with 150 CFU *M.tb* (Erdman strain) by aerosol inhalation. 23 days following infection the lungs and spleen were removed for analysis. Representative organs were imaged from each genotype prior to n=2 tissues per genotype being embedded for histochemical analysis. Sections were cut from all tissues and subjected to anti-Galectin 3 (mac2 epitope) immunohistochemistry to detect macrophages within granuloma (Galectin 3 – red, nuclei - blue). Scale bar 50µm.

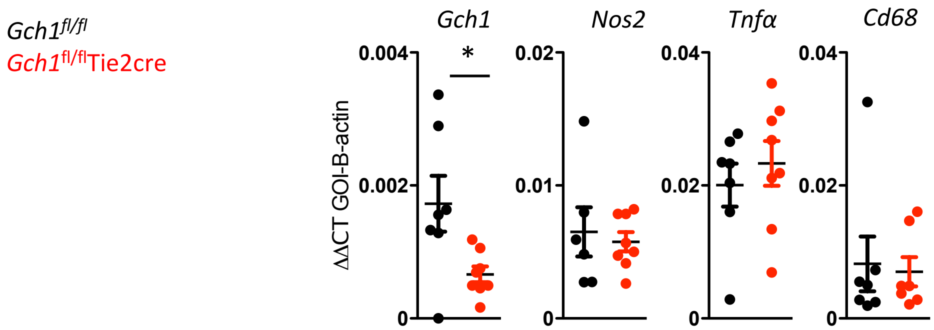

Supplementary Figure 7: *Gch1*$^{fl/fl}$Tie2cre mice exhibit significantly less *Gch1* expression in *M.tb* infected lungs but show no alteration in *Nos2, Tnfα or Cd68* gene expression. RNA was produced from *M.tb* infected lungs harvested 6 weeks after infection with $5x10^6$ cfu/ml *M.tb* Erdman strain by aerosol inhalation. Whole lungs were homogenised for CFU measurement and RNA was prepared from excess homogenate and quantitative real-time PCR was used to quantify *Gch1 , Tnfα, Cd68* and *Nos2* gene expression (n= 7-8 per group, $p<0.05$, error bars +/- SEM).

a

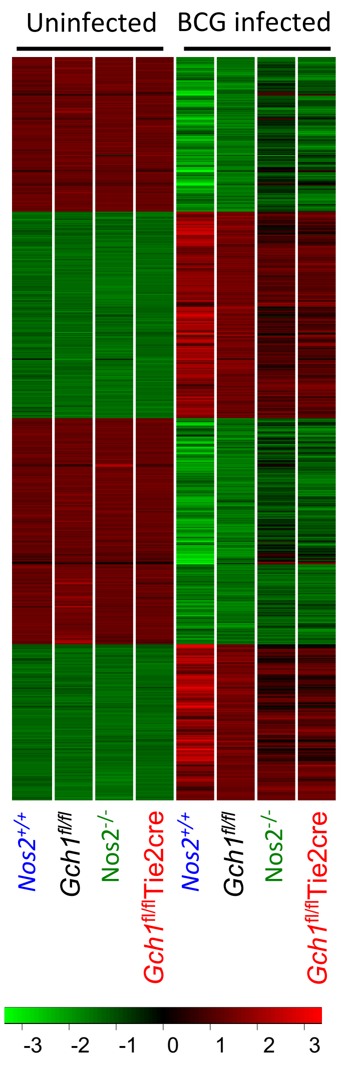

b

Categories	Diseases or Functions Annotation	p-Value	Predicted Activation State	Activation z-score
Cell-To-Cell Signaling and Interaction, Immune Cell Trafficking	activation of leukocytes	3.46E-19	Increased	4.583
Antimicrobial Response, Inflammatory Response	antimicrobial response	6.52E-16	Increased	4.031
Cell Death and Survival, Cellular Compromise	cytotoxicity of leukocytes	4.37E-10	Increased	3.565
Cell-To-Cell Signaling and Interaction, Immune Cell Trafficking	recruitment of leukocytes	9.22E-19	Increased	3.488
Cell-To-Cell Signaling and Interaction, Inflammatory Response	immune response of leukocytes	2.74E-18	Increased	2.757

c

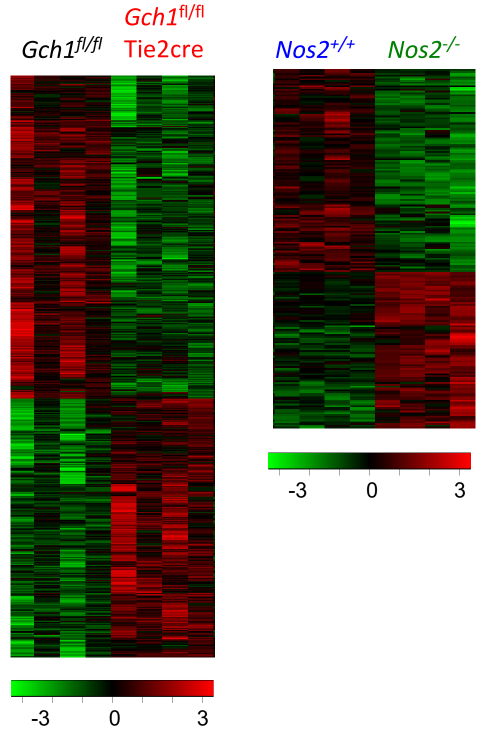

Supplementary Figure 8: Absence of *gch1* or *nos2* modulates macrophage gene expression in response to infection. Macrophages were infected with BCG (MOI 1:1)/ IFNγ (10ng/ml) for 24 hours or incubated for 24hrs in the absence of infection (n=4 per genotype). RNA was extracted and a whole mouse genome gene expression array was performed. Genes were determined as significantly regulated by infection or genotype by comparison to the untreated or wildtype control for adj p<0.05. **(a)** Heat map showing gene expression of the top 1000 genes significantly regulated (adj p<0.05) by BCG infection in wildtype macrophages, ranked by fold difference between uninfected and BCG infected BMDM. Data shown are the average normalized log2 expression value for each group normalized to the expression across all samples. Green represents a negative change and red represents a positive change. **(b)** Selected relevant gene functional annotations significantly regulated in wildtype macrophages by BCG infection, as determined using IPA analysis with Z score >2 and a p-value for overlap p<0.01. **(c)** Heat map showing gene expression of all genes significantly regulated (adj p<0.05) for BCG infected macrophages, ranked by fold difference between control and *Gch1*$^{fl/fl}$Tie2cre or *Nos-/-* macrophages.

a

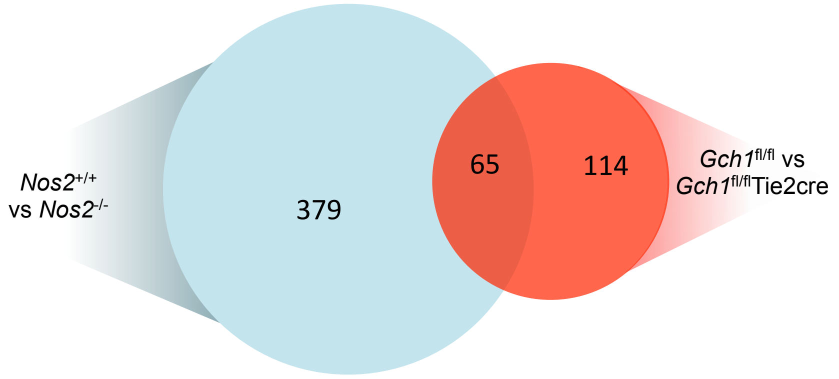

b

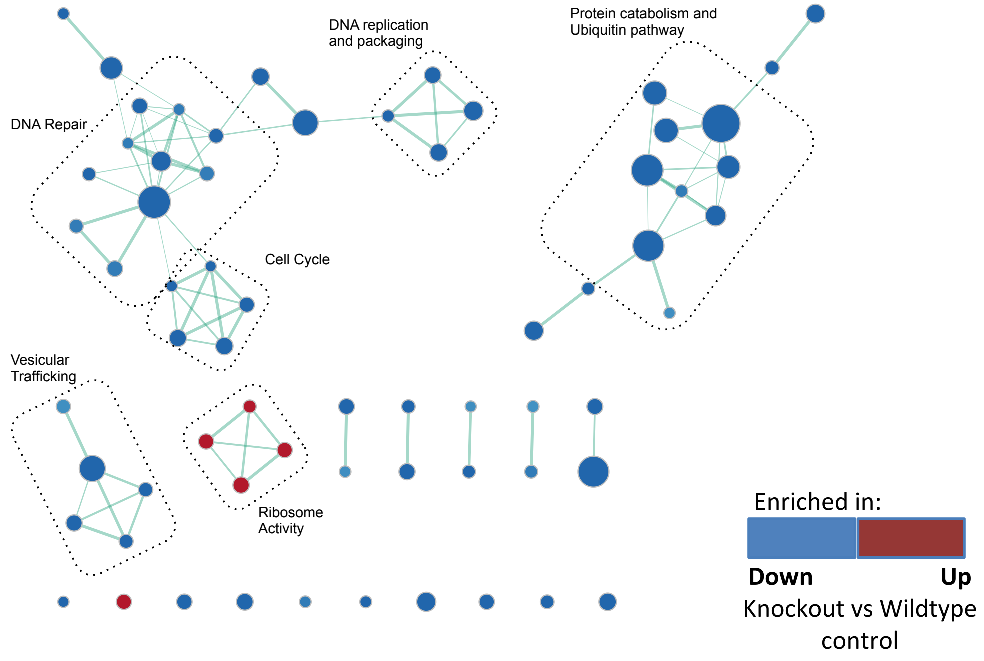

Supplementary Figure 9: Gene set enrichment using GO term functional annotations identifies shared gene expression patterns in BCG/IFNγ treated macrophages. Enriched gene sets in BCG infected macrophages, identified by GSEA (MSigDb, C5 GO BP annotations set). To determine how far regulated gene functions were shared between the *Gch1* and *Nos2* knockout macrophages the gene sets passing $p<0.005$, FDR $q<0.1$ in both analyses were compared (a) the genesets present in both were used to plot an Enrichment Map in Cytoscape (b).

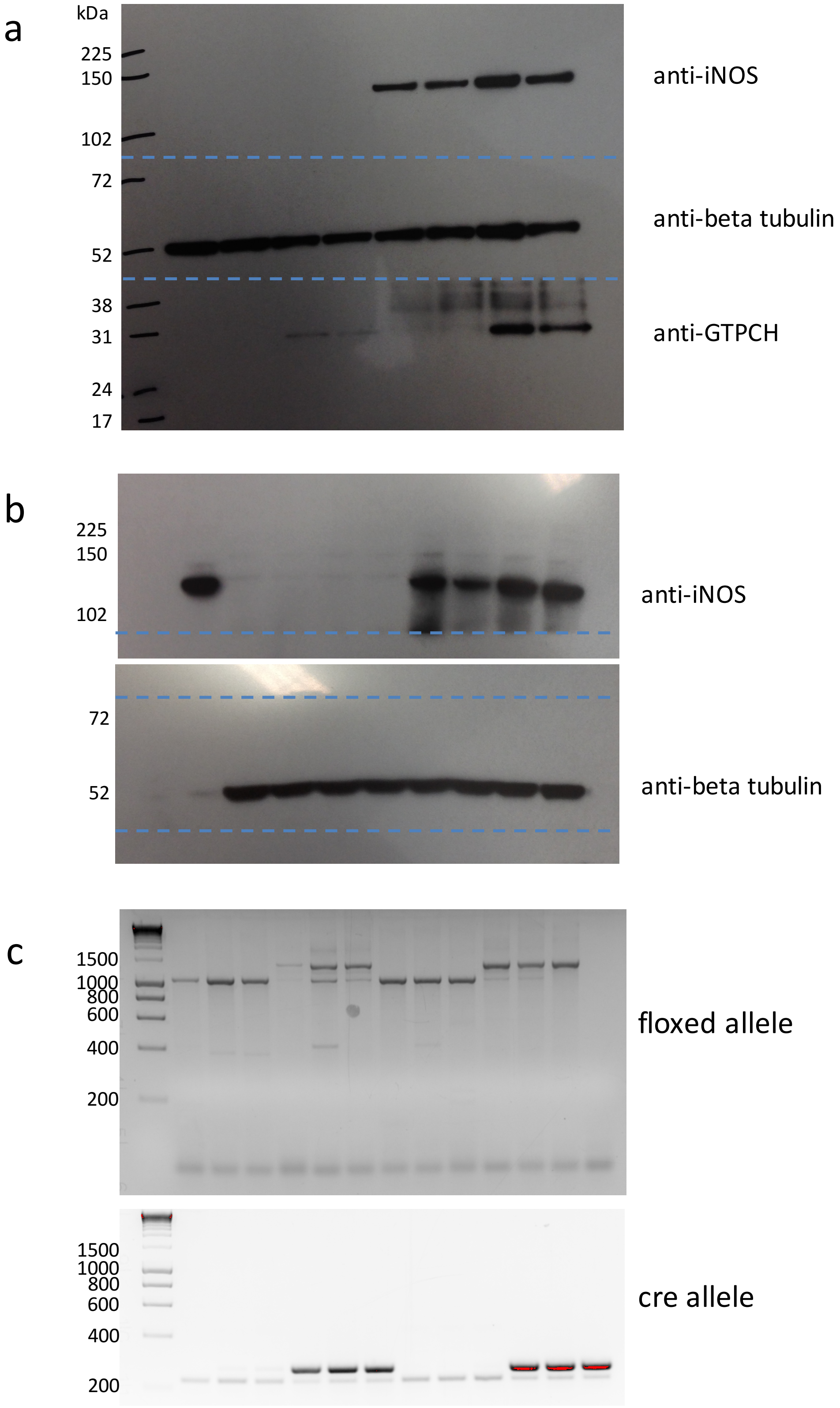

Supplementary Figure 10: Source Data. (a) Source data for Figure 1b. **(b)** Source data for Figure 2i. **(c)** Source data for Figure 2a. Western membranes in a and b were cut at the blue dotted point after transfer for parallel incubation of regions with the indicated antibodies.

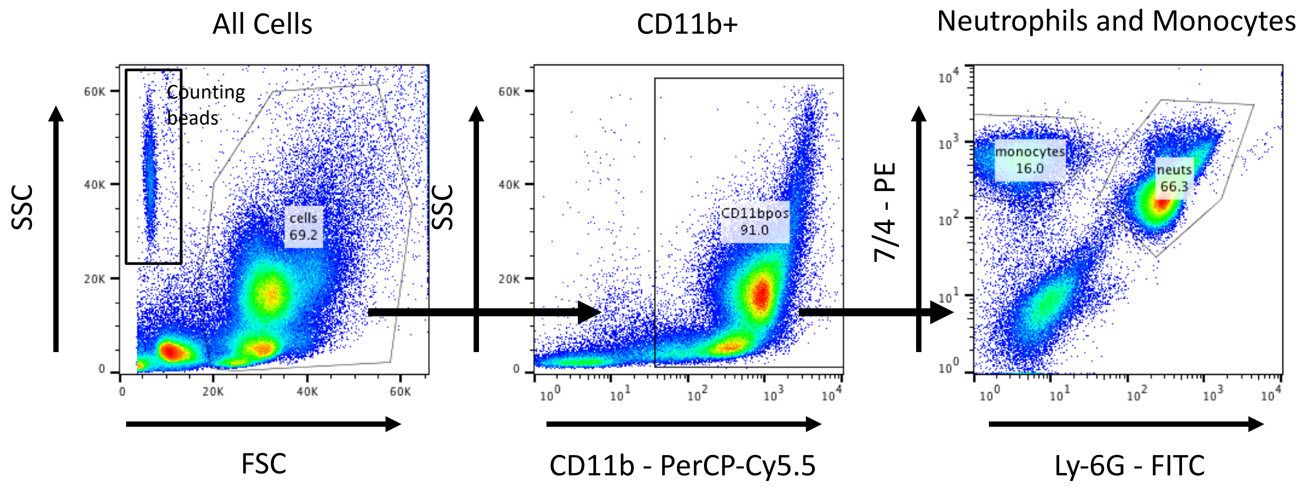

Supplementary Figure 11: Gating Scheme. (a) Example gating scheme to accompany Figure 1h.
