# Supplementary material for: Regulation of mycobacterial infection by macrophage Gch1 and tetrahydrobiopterin
Source: Nat Commun. 2018 Dec 20;9:5409. doi: 10.1038/s41467-018-07714-9 (PMC6302098; doi:10.1038/s41467-018-07714-9)
Supplement: Supplementary file 3 — Description of Additional Supplementary Files [file 41467_2018_7714_MOESM3_ESM.pdf]

Description of Additional Supplementary Files

Supplementary Data 1

Description: Differentially expressed genes in response to BCG/IFNγ in wildtype macrophages. Genes significantly regulated (adj $p<0.05$) in wildtype BMDM uninfected vs BCG IFNγ infected. A key to the table is provided in a separate spreadsheet tab.

Supplementary Data 2

Description: Selected functional annotations significantly modulated in infected wildtype macrophages using Ingenuity Pathway Analysis.

Supplementary Data 3

Description: Differentially expressed genes by genotype in uninfected or BCG/IFNγ infected macrophages. Genes significantly regulated (adj $p<0.05$) *Gch*$^{fl/fl}$ vs *Gch*$^{fl/fl}$*Tie2cre* and *Nos2*$^{+/+}$ vs *Nos2*$^{-/-}$. A key to the table is provided in a separate spreadsheet tab.

Supplementary Data 4

Description: Cellular functions significantly modulated in infected *Gch*$^{fl/fl}$*Tie2cre* macrophages using Ingenuity Pathway Analysis.

Supplementary Data 5

Description: Upstream regulators significantly modulated in infected *Gch*$^{fl/fl}$*Tie2cre* macrophages using Ingenuity Pathway Analysis.

Supplementary Data 6

Description: Significantly altered gene sets in infected macrophages due to loss of *Gch1* or *Nos2*. A key to the table is provided in a separate spreadsheet tab.
