# Supplementary material for: Regulation of mycobacterial infection by macrophage Gch1 and tetrahydrobiopterin
Source: Nat Commun. 2018 Dec 20;9:5409. doi: 10.1038/s41467-018-07714-9 (PMC6302098; doi:10.1038/s41467-018-07714-9)
Supplement: Supplementary file 5 — Supplementary Dataset 2 [file 41467_2018_7714_MOESM5_ESM.pdf]

Selected functional annotations significantly modulated in infected wildtype macrophages using Ingenuity Pathway Analysis

Categories	Diseases or Functions Annotation	p-Value	Predicted Activation State	Activation z-score	Molecules
Antimicrobial Response	antimicrobial response	6.52E-16	Increased	4.031	ADAR,AIM2,ANXA3,APOBEC3B,BAK1,BANF1,BCL3,BIRC2,BIRC3,BNIP3,Bst2,CCL5,CD40,CD86,CEBPB,CLEC4E,COTL1,CXCL10,Cxcl9,CXCR4,DDX3X,DDX58,DDX60,DHX58,EIF2AK2,FCGR1A,HCK,HLA-A,HLA-E,HLA-G,HSPA8,IFI16,IFI44,IFIH1,IFIT1,IFIT1B,IFIT2,IFNGR1,IL12RB1,IL15,IL17RA,IL1B,IL23R,IL27,IL6,IRF1,IRF5,IRF7,IRF8,ISG15,ISG20,IVNS1ABP,MB21D1,MMP12,Mx1/Mx2,MYD88,NAIP,NCF1,NLRP3,NOD1,NOD2,NOS2,OAS1,OAS3,OASL,PLD1,PPIA,PRKRA,PTPN22,RICTOR,RNASEL,RSAD2,SAMHD1,SERPINB9,SLAMF8,SLC11A1,SOCS1,SOCS3,SPRY2,STAB1,STAT1,STAT2,SYK,TAGAP,TBK1,TICAM2,Tlr11,TLR3,TLR6,TLR9,TNF,TNFRSF1A,TRIM5,TRIM56,UNC13D,USP25,VAV1,ZC3H12A,ZC3HAV1
Cell Death and Survival	cell viability of leukocytes	5.18E-09	Increased	3.408	AKT1,AKT2,BAK1,BCL2A1,BCL3,C3,CARD11,CD28,CD300A,CD38,CD40,CD44,CD47,CD81,CD86,CEBPB,CHEK2,CSF1,CSF1R,CXCR4,CYLD,FAS,FCGR1A,FCGR3A/FCGR3B,FOXO1,HCK,HIF1A,HSH2D,ICAM1,ICOSLG/LOC102723996,IL15,IL18,IL1B,IL21R,IL27,IL6,IL7R,ITGAL,JAK2,LAT,LYN,MAP2K1,MAPK3,MAPK7,MEF2C,MYC,MYD88,NFIL3,NFKB1,NFKBIA,NME1,PARP14,PIK3CG,PIM1,PIM2,PML,REL,RELB,RHOA,RICTOR,RPTOR,Saa3,SCARB1,SERPINB9,SH3KBP1,SOD2,STAT1,STAT3,SYK,TAF4B,TGFB1,TGM2,TLR3,TLR9,TNF,TNFSF10,TNFSF15,TRAF2,TRAF3,TYK2
	apoptosis of macrophages	1.64E-12	Increased	2.283	AKT1,BIRC2,BIRC3,CASP1,CASP3,CASP4,CASP6,CCL5,CD14,CDKN1A,CEBPB,CFLAR,CSF1,CYBB,DDIT3,DFFA,EIF2AK2,FAS,GPR132,HIF1A,IL18,IL1B,IRF1,IRF2,ISG15,LRP1,MAPK14,MAPK9,MYD88,NAMPT,NCEH1,NFKBIA,NOS2,PLA2G4A,PPARG,PRKCE,SOD2,ST6GAL1,STAT1,STAT3,TGFB1,TICAM2,TNF,TNFRSF1A,TNFSF10
	cytotoxicity of leukocytes	4.37E-10	Increased	3.565	ARL8B,CARD11,CASP1,CCL2,CCL5,CD226,CD274,CD300A,CD38,CD44,CD69,CD81,CEACAM1,CFLAR,CYBB,FAM49B,FAS,FCGR1A,FCGR3A/FCGR3B,HAVCR2,HCK,HLA-A,HLA-E,HLA-G,HSPA1A/HSPA1B,HSPA8,ICAM1,IGF1,IL15,IL18,IL21R,IL27,IL6,ITGAL,Klrk1,LAT,LYN,MYD88,NFKB1,NOS2,PNP,SLAMF6,SOCS1,STAT1,SYK,TAP2,TGFB1,TLR9,TNF,TNFSF10,TNFSF12,Tnfsf9,TXN,VAV1
Cell Morphology	autophagy	1.08E-11	Increased	2.454	ACLY,ACP2,ACSL1,AKT1,AKT2,AKT3,ARSB,ATF4,ATG4C,BIRC2,BNIP3,C19orf12,C9orf72,CAMK1,CASP1,CAT,CCL2,CD38,CD4,CDKN1A,CEBPB,CFLAR,CHMP4B,CISD1,CISD2,CTSD,CXCR3,CXCR4,CYB5A,CYBB,DDIT3,DEPTOR,DRAM1,DUSP4,E2F1,EEF2K,EIF2AK2,EIF2AK3,FAS,FBXO7,FKBP1A,FOXO1,GAB1,Gm21596/Hmgb1,GNAS,GPR18,HERC1,HGS,HIF1A,HIVEP2,HMOX1,HSPA8,IDO1,IFNGR1,IGF1,IGF1R,Igtp,Iigp1,IL1B,IL6,Irgm1,ITPR1,KAT8,KDR,LARP1,MAP1LC3A,MAP1LC3B,MAP2K1,MAPK14,MCOLN3,MFSD8,MTDH,MYC,MYD88,NAMPT,NCF1,NOD2,NOS2,NUPR1,PDK2,PIM2,PINK1,PIP4K2A,PLA2G4A,PLD1,PPARG,PRNP,PTPN22,RAC3,RNASEL,RPTOR,RUBCN,SCD,SESN2,SH3BP4,SH3GLB1,SIRT6,SMYD3,SOD2,SOGA1,SPP1,SPTSSA,SQSTM1,SREBF2,STAT1,STX17,TBC1D4,TBC1D9,TBK1,TECPR1,TGFB1,TICAM2,TLR1,TLR3,TLR6,TLR9,TMBIM6,TMEM59,TMEM74,TNF,TNFSF10,TPCN1,TRAF2,TRIB3,TRIM21,TRIM5,ULK2,VPS25,VPS36,VPS37B,WDR45,ZC3H12A
Cell Signaling	synthesis of nitric oxide	5.42E-11	Increased	2.203	ADORA2A,ADRB2,AIF1,AKT1,AKT2,CASP1,CAT,CD14,CD28,CD38,CD40,CEBPB,CNP,CNR2,CSF1,CYBB,DDAH2,EDN1,ESR1,FASN,FKBP1B,FLT1,GCH1,HCK,HDC,HLA-G,HMGCR,HMOX1,ICAM1,IFNGR1,IGF1,IL15,IL18,IL1A,IL1B,IL1RN,IL6,ILK,INSR,IRF1,IRF8,ITGB1,JAK2,JUNB,KLF4,KLF6,Klrk1,LRP1,MAP2K1,MAP3K1,MAPK14,MAPK9,MYD88,NCF1,NFKBIA,NOS2,NR3C1,PARP1,PIK3CG,PKD2,PLAU,PRKCE,PTGS2,PTK2,RIPK2,RPSA,S100A1,S1PR1,SCARB1,SLC7A2,SOCS1,SOCS3,SOD2,SPP1,STAT1,TFPI,TGFB1,TLR6,TLR9,TNF,TNFAIP3,TNFRSF1A,TNFSF10,TRAF2,TRAF3,TSPO,TYK2,VEGFA,ZC3H12A
Cell-mediated Immune Response	differentiation of T lymphocytes	1.93E-14	Increased	3.147	AFF1,AKT1,ANXA1,BATF,BCL2A1,BCL3,Bhlhe41,BRAF,C5AR1,CARD11,CCL5,CD226,CD274,CD28,CD4,CD69,CD83,CD86,Cdkn1c,CEBPA,CEBPB,CFLAR,CHD7,CIITA,CLEC4E,CR1L,CXCR4,CYLD,DCLRE1C,DNMT1,E2F1,EGR1,EGR2,ELF4,ETS1,FCGR2B,FOXO1,GADD45B,GPR183,HAVCR2,HIVEP2,HLA-DMA,HLA-DOA,HLA-G,ICAM1,ICOSLG/LOC102723996,IFNGR1,IGF1,IGF1R,IGF2R,IL10RA,IL12RB1,IL15,IL17RA,IL18,IL1B,IL1RL1,IL21R,IL23R,IL27,IL4R,IL6,IL7R,IRAK3,IRF1,IRF2,IRF5,IRF8,ITGAL,JMJD6,JUNB,KDELR1,KLF4,LAT,LCP2,LFNG,LYL1,MAFB,MAP2K1,MAPK14,MAPK9,MARCH1,MBP,MERTK,MLLT10,MYD88,NDFIP1,NOS2,PARP1,PATZ1,PIK3CG,PNP,PPARG,PTGS2,PTPN2,PTPN22,REL,RELB,RFTN1,RHOA,RHOH,RICTOR,RIPK2,RPL22,RSAD2,S1PR1,SEMA4A,SLAMF6,SLC3A2,SOCS1,SOCS3,ST3GAL5,STAT1,STAT2,STAT3,SYK,TCF12,TGFB1,TGFBR1,TNF,TNFAIP3,Tnfsf9,TYK2,USP18,VAV1,WWP1,ZBTB16,ZBTB7B
Cell-To-Cell Signaling and Interaction	recruitment of leukocytes	9.22E-19	Increased	3.488	ACVRL1,AKT2,ALOX5AP,ANXA1,BCL3,BRAF,C3,C5AR1,CAMK1,CASP1,CAT,Ccl2,CCL2,CCL3L3,CCL5,Ccl7,CCR2,CD14,CD274,CD28,CD300LB,CD37,CD4,CD40,CD44,CD47,CD69,CD93,CD99L2,CLEC4E,CNR2,CSF1,CSF1R,CTSC,CXCL10,CXCL16,CXCL3,Cxcl9,CXCR3,CXCR4,DDX58,EDN1,EGR1,ETS1,FCGR2B,FCGR3A/FCGR3B,FLT1,FPR2,GCNT1,Gm21596/Hmgb1,GSN,HCAR2,HCK,HDC,HIF1A,HMOX1,HSPA1A/HSPA1B,ICAM1,IDO1,IL15,IL15RA,IL17RA,IL18,IL1A,IL1B,IL1RL1,IL1RN,IL21R,IL6,IRF5,IRF7,KDM6B,KDR,LAMTOR2,LRP1,LSP1,LYN,MAPK14,MAPK7,MYD88,NFKBIA,NINJ1,NLRP3,NOD1,NOD2,NRAS,PARP1,PDE4B,PELI1,PIK3CG,PIP5K1C,PLGRKT,PPARG,RCAN1,RHOA,RHOB,RIPK2,RTN4,RXRA,S100A4,S1PR1,SELL,SLFN12L,SOCS1,SOD2,SPP1,ST3GAL4,STAT3,SYK,TFPI,TGFB1,THBS1,TLR3,TLR9,TNF,TNFRSF1A,TNFSF12,TREML2,TRPM2,TXN,VAV1,VAV3
	activation of leukocytes	3.46E-19	Increased	4.583	ABHD12,ADORA2A,AHNAK,AKT1,ANXA1,ANXA2,AZI2,BAK1,BCL3,C3,C5AR1,CARD11,CASP1,CCDC88B,CCL2,Ccl2,CCL5,CCR2,CD14,CD226,Cd24a,CD274,CD28,CD300A,CD300LB,CD37,CD38,CD4,CD40,CD44,CD47,CD63,CD69,CD81,CD83,CD84,CD86,CD93,CDKN1A,CEACAM1,CEBPB,CERK,CFH,CLCN7,CLEC4E,Cmah,CNR2,CR1L,CRY1,CSF1,CSF1R,CST3,CTSH,CXCL10,CXCL3,CYBB,DDIT3,DDOST,DDX58,DUSP3,EDN1,EGR2,ETS1,FAM49B,FAS,FCGR1A,FCGR2B,FCGR3A/FCGR3B,FKBP1A,FOXO1,FPR1,FPR2,Gm21596/Hmgb1,GPNMB,HAVCR2,HCK,HDAC5,HDAC9,HIF1A,HLA-A,HLA-DMA,HLA-DMB,HLA-DOA,HLA-DQB1,HLA-G,HMOX1,HRH2,HSH2D,ICA1,ICAM1,ICOSLG/LOC102723996,IDO1,IGF1,IGF2R,IL12RB1,IL15,IL15RA,IL18,IL1A,IL1B,IL1RL1,IL1RN,IL21R,IL27,IL4R,IL6,IRAK3,IRF1,IRF5,IRF8,ITGAL,ITGB1,JAK2,JMJD6,KLF4,Klrk1,LAT,LCP2,LRP1,LYN,MAP2K1,MAPK14,MAPK3,MAPK9,MBP,MERTK,Milr1,mir-29,MR1,MYD88,NCF1,NDFIP1,NFIL3,NFKB1,NFKB2,NFKBIA,NFKBIZ,NLRC3,NOD2,NOS2,NR1H3,PARP1,PDCD1LG2,PELI1,PIK3CG,PILRB,PPIA,PRKCE,PRKCI,PRNP,PSMB8,PSMB9,PSME2,PTGES,PTGS2,PTPN22,RAB29,RAB32,RAB34,RASGRP1,REL,RELB,RHOA,RHOB,RHOH,RIPK2,Saa3,SBNO2,SELL,SEMA4A,SERPINB9,SERPING1,SIAE,SLAMF6,SLC11A1,SLC7A2,SOCS1,SPP1,ST6GAL1,STAT1,STAT3,STX11,SYK,TARM1,TGFB1,TGM2,THBS1,TLR1,TLR3,TLR9,TNF,TNFAIP3,TNFRSF1A,TNFRSF21,TNFSF10,TNFSF12,TRAF2,TRAF3,TREM2,TREML2,TRPM2,TSPAN32,TYK2,UNC13D,USP18,VAV1,VAV3,VCAN,VEGFA,ZBTB7B
	immune response of leukocytes	2.74E-18	Increased	2.757	ADORA2A,ANXA1,ANXA5,BAK1,BRD2,C3,C5AR1,CAPG,CARD11,CASP1,Ccl2,CCL2,CD14,CD226,CD274,CD28,CD38,CD40,CD44,CD47,CD69,CD86,CD93,CEBPB,CFH,CH25H,CLEC9A,CMC2,CST3,CXCL10,CXCL3,DDX58,ETS1,FAS,FCGR1A,FCGR2B,FCGR3A/FCGR3B,FOXO1,FPR1,Gm21596/Hmgb1,GNAS,GPR18,GSN,HAVCR2,HCK,HLA-DQB1,HMOX1,ICAM1,ICOSLG/LOC102723996,IFNGR1,IL10RA,IL12RB1,IL15,IL18,IL1B,IL1RAP,IL1RL1,IL23R,IL6,IL7R,IRF2,IRF8,ISG15,ITGAL,ITGB1,ITGB3,JMJD6,LCP2,LYN,MAPK14,MARCH1,MARCO,MERTK,MSH2,MYD88,NAMPT,NOD2,NOS2,NPM1,NR3C1,PARP1,PDCD1LG2,PIK3CG,PLAU,PNP,PPARG,PSMB8,PSME2,RAB43,RASA4,REL,RELB,Rfx5,SCARB1,SCARF1,SEMA4A,SERPINB9,SIRPB1,SLC1A5,SOCS1,SOCS3,STAT3,SYK,TAP1,TAPBP,TGFB1,TGM2,THBD,THBS1,TLR3,TLR9,TNF,TNFRSF1A,TREM2,TREML2,TREX1,TYK2,VAV1

Continues on next page

Categories	Diseases or Functions Annotation	p-Value	Predicted Activation State	Activation z-score	Molecules
Continued					
Cellular Development	leukopoiesis	1.45E-18	Increased	3.503	ADCY7,AFF1,AKT1,AKT2,ANXA1,ARID5B,AZI2,B4GALNT1,BATF,BATF2,BCL2A1,BCL3,Bhlhe41,BRAF,BST1,C3,C5AR1,CARD11,CASP1,CCL2,CCL5,CCND2,CCR2,CD14,CD226,Cd24a,CD274,CD28,CD300A,CD38,CD4,CD40,CD44,CD47,CD69,CD81,CD83,CD86,CD9,CDK2,CDKN1A,Cdkn1c,CEACAM1,CEBPA,CEBPB,CFLAR,CHD7,CIITA,CLEC4E,CNR2,CR1L,CRTC2,CSF1,CSF1R,CSF3R,CST3,CXCL10,CXCL3,CXCR3,CXCR4,CYLD,DCLRE1C,DCSTAMP,DDX58,DNMT1,E2F1,EED,EGR1,EGR2,ELF4,EMP1,ESR1,Esrra,ETS1,ETV6,FAS,FASN,FCGR1A,FCGR2B,FKBP1A,FLT1,FNIP1,FOXO1,GADD45B,GAS6,GCNT1,GPR183,HAVCR2,HCLS1,HDAC5,HDAC9,HELLS,HIF1A,HIVEP2,HLA-A,HLA-DMA,HLA-DOA,HLA-DQB1,HLA-G,ICAM1,ICOSLG/LOC102723996,IFI16,IFNGR1,IFRD1,IGF1,IGF1R,IGF2R,IL10RA,IL12RB1,IL15,IL15RA,IL17RA,IL18,IL1A,IL1B,IL1RL1,IL1RN,IL21R,IL23R,IL27,IL4R,IL6,IL7R,INF2,IRAK3,IRF1,IRF2,IRF5,IRF7,IRF8,ITGAL,ITGB3,ITM2A,JAK2,JMJD6,JUNB,KDELR1,KDR,KLF4,Klrk1,LAT,LCP2,LFNG,LGALS8,LMO2,LSP1,LYL1,LYN,MAFB,MAP2K1,MAPK14,MAPK3,MAPK9,MARCH1,MBP,MEF2C,MERTK,MLLT10,MMP14,MSH2,MYC,MYD88,NBN,NDFIP1,NFAM1,NFIL3,NFKB1,NFKB2,NFKBIA,NFKBIZ,NOS2,NQO2,NRP1,PARP1,PATZ1,PDCD1LG2,PIK3CG,PIM1,PLA2G4A,PLCL2,PML,PNP,PPARG,PRKCH,PRNP,PSMB8,PSMB9,PSTPIP1,PTGS1,PTGS2,PTPN2,PTPN22,RALA,RALGDS,RASGRP1,RASGRP3,RBL1,REL,RELB,RFTN1,RGL2,RGS10,RHOA,RHOH,RICTOR,RIPK2,RPL22,RPTOR,RSAD2,S1PR1,SEMA4A,SENP1,SIRT6,SLAMF6,SLC19A1,SLC3A2,SOCS1,SOCS3,SPP1,SPPL2A,ST3GAL5,STAT1,STAT2,STAT3,SYK,TAPBP,TCF12,TGFB1,TGFBR1,THEMIS2,TLR1,TLR3,TLR6,TLR9,TNF,TNFAIP3,TNFRSF1A,TNFSF10,TNFSF15,Tnfsf9,TRAF2,TRAF3,TREM2,TRPM2,TYK2,USP18,VAV1,VAV3,VEGFA,WWP1,ZBTB16,ZBTB7B
	maturation of phagocytes	2.87E-09	Increased	2.851	AES,CCL5,CCR2,CD40,CD44,CD47,CD83,CD86,CSF1,E2F1,EGR1,FAS,FCGR2B,Gm21596/Hmgb1,HAVCR2,HMOX1,HSPA1A/HSPA1B,ICAM1,IFIH1,IL15,IL1A,IL1B,IL6,IRF1,IRF8,LYN,MAP2K6,MYD88,NFKB2,NUP98,PML,RELB,RHOA,STAT1,STAT3,TGFB1,TGFBR1,THBS1,Tlr11,TLR3,TLR9,TNF,TNFRSF1A,TNFSF10,TREM2
Cellular Function and Maintenance	cellular homeostasis	8.34E-19	Increased	3.425	ABCB7,ACLY,ACO1,ACOT11,ACP2,ACSL1,ADCY7,ADIPOR1,ADORA2A,ADRB2,AFF1,AKR1B1,AKT1,AKT2,AKT3,ANXA1,ANXA2,ANXA6,AP2A2,APLP2,ARFGEF3,ARHGEF7,ARSB,ATF4,ATG4C,ATP1A3,ATP1B3,ATPIF1,ATXN3,BAK1,BATF,BCL2A1,BCL3,BCO2,Bhlhe41,BIRC2,BNIP3,BRAF,C19orf12,C3,C5AR1,C9orf72,CACNA1A,CAMK1,CARD11,CASP1,CASP3,CASP7,CAT,CCL2,Ccl2,CCL3L3,CCL5,CCR2,CD226,Cd24a,CD274,CD28,CD300A,CD38,CD4,CD40,CD47,CD69,CD81,CD83,CD86,CDK2,CDKN1A,Cdkn1c,CEACAM1,CEBPA,CEBPB,CFLAR,CHD7,CHEK2,CHMP4B,CHRM3,CIITA,CISD1,CISD2,CKB,CLCN5,CLEC4E,CLIC5,CLSTN1,CNP,CNR2,CP,CR1L,CRACR2B,CRTC2,CRY1,CTSD,CTTN,CXCL10,CXCL3,CXCR3,CXCR4,CYB5A,CYBB,CYCS,CYLD,DCLRE1C,DCXR,DDIT3,DEPTOR,DFFA,DNMT1,DOCK11,DRAM1,DUSP4,E2F1,EDN1,EED,EEF2K,EGR1,EGR2,EIF2AK2,EIF2AK3,ELF4,ENPP1,ERC1,ERO1A,ESR1,Esrra,ETS1,FAM3B,FAS,FBXO7,FCGR1A,FCGR2B,FCGR3A/FCGR3B,FECH,FKBP1A,FKBP1B,FLT1,FMO5,FNIP1,FOXO1,FPR1,FPR2,GAB1,GADD45B,GAPT,GAS6,GDF3,Gk,Gm21596/Hmgb1,GNA12,GNA13,GNAQ,GNAS,GPD2,GPR132,GPR18,GPR183,GSN,HADH,HAVCR2,HCK,HDC,HEBP1,HECTD4,HELZ2,HERC1,HEXB,HFE,HGS,HIF1A,HIVEP2,HLA-A,HLA-DMA,HLA-DOA,HLA-DQB1,HLA-G,HMOX1,HSPA8,ICA1,ICAM1,ICOSLG/LOC102723996,IDO1,IFNGR1,IGF1,IGF1R,IGF2R,IGFBP4,Igtp,Iigp1,IL10RA,IL12RB1,IL15,IL15RA,IL17RA,IL18,IL1A,IL1B,IL1RL1,IL1RN,IL21R,IL23R,IL27,IL4R,IL6,IL7R,INSR,IRAK3,IRF1,IRF2,IRF5,IRF8,Irgm1,ITGAL,ITGB1,ITGB3,ITPR1,JAK2,JMJD6,JUNB,KAT8,KCNJ2,KDELR1,KDR,KLF4,Klrk1,L1CAM,LARP1,LAT,LCP2,LFNG,LIPE,LMO2,LPAR1,LRP5,LXN,LYL1,LYN,MAFB,MAFG,MAP1LC3A,MAP1LC3B,MAP2K1,MAPK14,MAPK3,MAPK9,MARCH1,MBP,MCOLN3,MEF2C,MERTK,MFSD8,MLLT10,MSRB2,Mt1,MTDH,MTHFD1,MTM1,MYADM,MYC,MYD88,NAMPT,NBN,NCF1,NCOA4,NDFIP1,NEDD4L,NFIL3,NFKB1,NFKBIA,NFKBIZ,NOD2,NOS2,NR3C1,NUCKS1,NUPR1,OAS1,P2RY1,PARP1,PATZ1,PDCD1LG2,PDE4B,PDK2,PGM1,PIK3CG,PIM1,PIM2,PINK1,PIP4K2A,PKD2,PLA2G16,PLA2G4A,PLAU,PLCB2,PLCB3,PLCL2,PLD1,PNP,PPA2,PPARG,PPIA,PRCP,PRKCE,PRKCH,PRKCI,PRKG1,PRNP,PROCR,PSMB8,PSMB9,PTGS1,PTGS2,PTK2,PTPN2,PTPN22,PYGL,RAC3,RAP2C,RAPGEF2,RASGRP1,RASGRP3,RBL1,REL,RELB,REST,RFTN1,RGS2,RHOA,RHOH,RICTOR,RIPK2,RMDN3,RMI1,RNASEL,RPL22,RPTOR,RSAD2,RTN4,RUBCN,S1PR1,SCD,SCO1,SELENON,SEMA4A,SEPT9,SESN2,SH3BP4,SH3GLB1,SH3KBP1,SIDT2,SIGMAR1,SIK3,SIRT6,SKIL,SLAMF6,SLC11A1,SLC11A2,SLC17A5,SLC24A3,SLC2A1,SLC31A1,SLC31A2,SLC39A10,SLC39A13,SLC39A6,SLC39A8,SLC3A2,SLC40A1,SLC8B1,SLC9A7,SMYD3,SOCS1,SOCS3,SOD2,SOGA1,SPOP,SPP1,SPTSSA,SQSTM1,SREBF2,ST3GAL5,STAT1,STAT2,STAT3,STK39,STX17,SYK,TAPBP,TBC1D4,TBC1D9,TBK1,TCF12,TCF7L2,TECPR1,TF,TGFB1,TGFBR1,TGM2,THBD,THEMIS2,TICAM2,TIMP2,TJP2,TLR1,TLR3,TLR6,TLR9,TMBIM6,TMEM59,TMEM74,TMTC2,TNF,TNFAIP3,TNFRSF1A,TNFSF10,TNFSF15,Tnfsf9,TPCN1,TRAF2,TRAF3,TRIB3,TRIM21,TRIM37,TRIM5,TRPM2,TYK2,ULK2,USP18,VASP,VAV1,VAV3,VEGFA,VEGFB,VPS25,VPS36,VPS37B,WDR45,WWP1,XCR1,ZBTB16,ZBTB7B,ZC3H12A
	phagocytosis	7.11E-16	Increased	2.702	ABR,ADORA2A,ANXA1,ANXA3,ANXA5,C3,CAPG,CAT,Ccl2,CD14,CD38,CD4,CD44,CD47,CD93,CEBPB,CERK,CFH,CH25H,CKB,CORO1C,CSF1,CSF3R,CXCL10,CYBB,DOCK1,EHD1,EIF2AK2,FAS,FCGR1A,FCGR2B,FCGR3A/FCGR3B,FPR1,GAS6,Gm21596/Hmgb1,GPR18,GSN,HCK,HMOX1,HSPA8,ICAM1,ICOSLG/LOC102723996,IL15,IL1B,IL1RL1,IL6,IQSEC1,IRF7,IRF8,ISG15,ITGB1,ITGB5,JMJD6,LAT,LRP1,LYN,MAPK14,MARCO,MCOLN3,MERTK,MRC1,MYC,MYD88,MYO5A,NCF1,NOD1,NOD2,NPM1,NR1H3,NR3C1,PELI1,PIP5K1C,PLA2G4A,PLAU,PLD1,Pot1b,PPARG,PRKCE,PSMD4,PTK2,RAB31,RACK1,RALA,RASA4,RELB,RHOA,RUBCN,RXRA,SCARB1,SCARB2,SCARF1,SH3GLB1,SH3KBP1,SIRPB1,SLC11A1,SNX3,STX17,SYK,TBK1,TECPR1,TGFB1,TGM2,THBS1,TLR1,TLR3,TLR9,TNF,TREM2,TREML2,UNC13D,VAMP7,VAV1
Drug Metabolism	synthesis of prostaglandin E2	7.15E-09	Increased	2.989	AKR1B1,ANXA1,CASP1,CD14,CD40,CD83,CEBPB,CERK,CSF1,EDN1,FASN,FCGR2B,HPGD,IGF1,IL15,IL18,IL1A,IL1B,IL1RN,IL27,IL6,JAK2,MAP2K1,MAP3K1,MAPK14,MAPK3,MAPK9,NCF1,NFKB1,NOS2,PLA2G4A,PLAA,PPARG,PRKCI,PRNP,PTGES,PTGS1,PTGS2,RELB,S1PR1,SGPP1,SOCS1,STAT3,TGFB1,TLR3,TNF,TNFRSF1A,TNFSF10
Free Radical Scavenging	synthesis of reactive oxygen species	2.39E-12	Increased	2.353	ABCB10,ACOD1,AKR1B1,AKT1,AKT2,ALDH2,ANXA1,ANXA2,ARAP3,ATPIF1,BAK1,BNIP3,C3,C5AR1,CASP3,CAT,CCL5,CCR2,CD14,CD28,CD40,CD44,CD47,CDKN1A,CFB,CFH,COL18A1,CRTC2,CSF1,CST3,CTTN,CYBB,DBI,DDIT3,DECR1,DHCR24,DHODH,DOCK5,E2F1,EDN1,EIF2AK3,ERO1A,Esrra,ETS1,ETV6,FAS,FCGR3A/FCGR3B,FOXO1,FPR1,FPR2,GCH1,GNAS,GPX1,GSN,HCK,HK1,HK2,HK3,HMOX1,HSD17B10,HVCN1,ICAM1,IDH1,IGF1,IL18,IL1B,IL6,ILK,INSR,ITGA6,ITGB1,ITGB3,JAK2,LAT,LCP2,LYN,MAOA,MAP2K1,MAPK14,MMP14,MPRIP,MRC1,MSRB2,MTUS1,MYD88,NAMPT,NCF1,NFKBIA,NOD1,NOS2,NRAS,PDE2A,PIK3CG,PIM1,PINK1,PLA2G4A,PLAGL2,PLAU,PLCB2,PLCB3,PON2,PON3,PPARG,PPIA,PRCP,PRKCE,PRNP,PTGS1,PTGS2,RAC3,RACK1,RALGDS,RHOA,RRM2,RTN4,S100A6,SCARB1,SDHC,SESN1,SH3BP5,SIGMAR1,SLC2A1,SOCS3,SOD2,SPP1,SPRY2,SQSTM1,SREBF2,STAT3,SYK,TF,TGFB1,TGM2,TLR3,TLR9,TMBIM6,TNF,TNFRSF1A,TRAF2,TRAF3,TREML2,Trim30a/Trim30d,TRPM2,ULK2,UNC13D,VAV1,ZC3H12A

Continues on next page

Categories	Diseases or Functions Annotation	p-Value	Predicted Activation State	Activation z-score	Molecules
Continued					
Hematological System Development and Function	quantity of leukocytes	2.35E-25	Increased	2.223	Abcb1b,ADORA2A,AFF1,AKT1,AKT3,ALDH2,ARHGEF6,ARID5A,ARID5B,B4GALNT1,BAK1,BATF,BCL2A1,BCL3,Bhlhe41,BIRC2,BIRC3,BST1,Bst2,C3,C5AR1,C5AR2,CACNA1A,CALCRL,CARD11,CASP3,CCL2,Ccl2,CCL3L3,CCL5,Ccl7,CCR2,Cd24a,CD274,CD28,CD300A,CD38,CD4,CD40,CD44,CD47,Cd59a,CD69,CD81,CD83,CD84,CD86,CDKN1A,CEBPA,CEBPB,CENPX,CERK,CFH,CFLAR,CIITA,CLEC10A,CLEC4E,Cmah,CNR2,COMT,CSF1,CSF1R,CSF3R,CTSD,CXCL10,CXCL16,CXCR3,CXCR4,CYBB,CYLD,CYP27A1,DCLRE1C,DDIT3,DDX58,DNMT1,E2F1,EBI3,EGR1,EGR2,ELF4,ESR1,Esrra,ETS1,ETV6,FAM26F,FAS,FCGR1A,FCGR2B,Fcrls,FLT1,FNIP1,FOXO1,FPR1,FPR2,FYB,GADD45B,GALNT1,GAPT,GCNT1,Gm21596/Hmgb1,GNA12,GNA13,GNAS,GPR132,GPR18,GPR183,GSN,HAVCR2,HCK,HCLS1,HDC,HELLS,HEXB,HIF1A,HIVEP2,HLA-A,HLA-DMA,HLA-DQB1,HLA-G,HMOX1,ICAM1,ICOSLG/LOC102723996,IDO1,IFNGR1,IGF1,IGF1R,IGF2R,IL10RA,IL12RB1,IL15,IL15RA,IL17RA,IL18,IL18BP,IL1A,IL1B,IL1RL1,IL1RN,IL21R,IL23R,IL27,IL4R,IL6,IL7R,IRAK3,IRF1,IRF2,IRF5,IRF8,ISG15,ITGA6,ITGAL,ITGB3,ITPR1,JAK2,KIFAP3,KLF4,Klrk1,LAT,LCP2,LGALS8,LIG1,LIPG,LSP1,LTC4S,Ly6a (includes others),LYL1,LYN,Mamld1,MAPK14,MAPK3,MAPK7,MAPK9,MARCH1,MBP,MERTK,MMP12,MR1,MSH2,Mt1,MXD1,MYC,MYD88,NBN,NDFIP1,NEDD4L,NFAT5,NFIL3,NFKB1,NFKB2,NFKBIA,NFKBIB,NIPAL3,NLRC3,NLRC5,NLRP3,NOD2,NOS2,NPM1,NQO2,NR3C1,NUP98,PARP1,PATZ1,PDCD1LG2,PDE4B,PIK3CG,PILRA,PIM1,PIM2,PLA2G15,PLAU,PLCL2,PML,PNP,Pot1b,PPARG,PPIA,PRKCE,PRKCH,PRMT7,PRNP,PSMB10,PSMB8,PSMB9,PTGS2,PTK2,PTPN2,PTPN22,RAC3,RASGRP1,RASGRP3,REL,RELB,Rfx5,RGS10,RHOA,RHOH,RICTOR,RIPK2,RPTOR,S1PR1,SCARB1,SCARF1,SCMH1,SELL,SERPINB6,SH2D3C,SIAE,SIRT6,SLAMF6,SLC19A1,SLC2A1,SLC39A10,SLC6A6,Slfn1,SLFN12L,SOCS1,SOCS3,SPP1,SPPL2A,ST3GAL2,ST3GAL4,ST6GAL1,STAB1,STAT1,STAT3,STX11,SYK,TANK,TAP1,TAPBP,TBK1,TCF12,TF,TGFB1,TGFBR1,TGM2,THBD,THBS1,TIMP2,TLR3,TLR9,TNF,TNFAIP3,TNFRSF1A,TNFRSF21,TNFSF10,TNFSF12,TNFSF15,TNIP1,TRAF1,TRAF2,TRAF3,TREM2,Trim30a/Trim30d,TSPO,TYK2,VAV1,VAV3,VEGFA,ZBTB16,ZBTB7B,ZC3H12A,ZFP36
Humoral Immune Response	quantity of IgG1	3.84E-09	Increased	2.01	BATF,CARD11,Ccl2,CD274,CD28,CD37,CD40,CD81,CD83,CD86,CDKN1A,Cmah,CYLD,DUSP4,FCGR2B,FPR2,GADD45B,HLA-DOA,HLA-DQB1,ICOSLG/LOC102723996,IL21R,IL4R,LYN,MSH2,MYD88,NFIL3,NFKB1,NFKB2,NUP98,PPIA,PTGS2,PTPN22,Pvr,RASGRP3,REL,Rfx5,RSAD2,SIAE,SPPL2A,TANK,TBK1,TNF,TNFRSF1A,TNFRSF21,TNFSF10,VAV1,ZC3H12A
Inflammatory Response	immune response of cells	4.04E-24	Increased	3.197	Abcb1b,ABR,ADORA2A,AKT1,ANXA1,ANXA3,ANXA5,ATXN3,BAK1,BIRC2,BIRC3,BRAF,BRD2,C3,C5AR1,CAPG,CARD11,CASP1,CAT,CCL2,Ccl2,CD14,CD226,CD274,CD28,CD38,CD4,CD40,CD44,CD47,CD69,CD83,CD86,CD93,CDKN1A,CEACAM1,CEBPB,CERK,CFH,CH25H,CKB,CLEC9A,CMC2,CORO1C,CSF1,CSF3R,CST3,CTSD,CXCL10,CXCL3,CYLD,DDX58,DHX58,DOCK1,EHD1,EIF2AK2,EIF4EBP2,ETS1,FAS,FCGR1A,FCGR2B,FCGR3A/FCGR3B,FOXO1,FPR1,GAS6,Gm21596/Hmgb1,GNA12,GNA13,GNAS,GPR18,GSN,HAVCR2,HCK,HLA-A,HLA-DQB1,HLA-E,HMOX1,HRH2,HSPA8,ICAM1,ICOSLG/LOC102723996,IFIH1,IFNGR1,IL10RA,IL12RB1,IL15,IL18,IL1B,IL1RAP,IL1RL1,IL23R,IL6,IL7R,IQSEC1,IRF1,IRF2,IRF7,IRF8,ISG15,ITGAL,ITGB1,ITGB3,ITGB5,JMJD6,LAT,LCP2,LYN,MAPK14,MARCH1,MARCO,MERTK,mir-29,MMP25,MRC1,MSH2,MYC,MYD88,NAMPT,NCF1,NLRP3,NOD1,NOD2,NOS2,NPM1,NR3C1,PARP1,PDCD1LG2,PELI1,PIK3CG,PIP5K1C,PLA2G4A,PLAU,PLD1,PML,PNP,POLA1,Pot1b,PPARG,PRKCE,PRKRA,PRNP,PSMB8,PSMD4,PSME2,PTK2,RAB31,RAB43,RACK1,RALA,RASA4,REL,RELB,Rfx5,RHOA,RICTOR,SCARB1,SCARF1,SEMA4A,SERPINB9,SH3KBP1,SIRPB1,SLC11A1,SLC1A5,SMYD3,SNX3,SOCS1,SOCS3,SPRY2,STAT1,STAT3,SYK,TAP1,TAPBP,TGFB1,TGM2,THBD,THBS1,TLR1,TLR3,TLR9,TNF,TNFRSF1A,TNFSF10,TNFSF12,TOP2A,TRAF3,TREM2,TREML2,TREX1,TRIM21,TRIM25,TRIM37,TRIM47,TRIM5,TRIM56,TYK2,UNC13D,USP25,VAV1,VEGFA
Lipid Metabolism	biosynthesis of polyunsaturated fatty acids	7.14E-09	Increased	2.307	AKR1B1,ALOX5AP,ANXA1,C5AR1,CASP1,Ccl2,CCL5,CD14,CD4,CD40,CD83,CEBPB,CERK,COTL1,CSF1,CXCL3,DECR2,DEGS1,EDN1,FADS1,FADS2,FASN,FCGR1A,FCGR2B,FPR1,HMOX1,HPGD,IGF1,IL15,IL18,IL1A,IL1B,IL1RN,IL27,IL6,JAK2,LTA4H,LTC4S,LYN,MAP2K1,MAP3K1,MAPK14,MAPK3,MAPK9,MYD88,NAPEPLD,NCF1,NFKB1,NFKBIA,NOS2,PLA2G4A,PLAA,PPARG,PRKCI,PRNP,PTGES,PTGS1,PTGS2,RELB,S1PR1,SCD,SGPP1,SOCS1,STAT3,SYK,TGFB1,TLR3,TNF,TNFRSF1A,TNFSF10,VEGFA
Organismal Survival	morbidity or mortality	5.81E-18	Increased	2.028	ABCA2,ABCB10,Abcb1b,ABCB4,ABCB7,ABR,ACACA,ACADM,ACLY,ACVRL1,ADAM15,ADAR,ADCY7,ADD1,ADH5,ADORA2A,ADRB2,AES,AFF1,AIM2,AKNA,AKT1,AKT2,AKT3,ALG2,ANAPC2,ANKH,ANXA1,ANXA2,APBB2,APEX1,APLP2,APRT,ARAP3,ARID5A,ARID5B,ARIH2,ARL8B,ASL,ATF4,ATMIN,ATOH1,ATP1A3,ATP6AP1,ATXN2,ATXN3,B3GALNT1,B4GALNT1,BAK1,BATF2,BCL2A1,BCL3,BFAR,BIRC2,BIRC3,Bmyc,BRAF,BRCA2,BRD2,C1QBP,C3,C5AR1,CA5B,CACNA1A,CACNB1,CALCRL,CAP1,CASP1,CASP3,CASP4,CASP7,CBY1,CCL2,Ccl2,CCL3L3,CCND2,CCNE1,CCR2,CD14,CD226,CD274,CD28,CD2AP,CD300A,CD4,CD40,CD44,CD47,Cd59a,CDC7,CDK2,CDK2AP1,CDK4,CDKN1A,Cdkn1c,CEACAM1,CEBPA,CEBPB,CEBPD,CEBPG,CENPJ,CERK,CFB,CFH,CFLAR,CHEK2,CISD2,CKB,CLCN7,CLEC4E,CLNS1A,Cmah,CNN3,CNP,CNPY2,COX17,CPOX,CR1L,CREB3L2,CREB5,CREM,CRIM1,CSF1,CSF1R,CSRNP1,CST3,CTBP1,CTSD,CTTN,Cux1,CXCL10,Cxcl11,CXCL3,Cxcl9,CXCR3,CXCR4,CYBB,CYCS,CYLD,CYP51A1,CYSLTR2,DAB2,DAG1,DAXX,DBI,DCLRE1C,DDB1,DDX3X,DDX58,DGKD,DHCR7,DHRS3,DHX58,DNMT1,DOCK1,DPAGT1,Dst,DTL,E2F1,E2F5,EDN1,EEF2K,EGR2,EHD1,EIF2AK2,EIF2AK3,ELOVL1,EP400,ERC1,ERCC1,ERO1A,ESR1,ETHE1,ETS1,ETV6,EXO1,EXT2,EYA1,FADS2,FAH,FANCM,FAP,FAS,FASN,FBN1,FCGR2B,Fcna,FDFT1,FECH,FH,FIG4,FKBP1A,FKBP1B,FKBP4,FKTN,FLNB,FLT1,FLVCR1,FOXO1,FPR1,FPR2,GAB1,GALNT1,GAMT,GAN,GAS6,GCH1,GDF3,GGCX,Gk,Gm21596/Hmgb1,GNA12,GNA13,GNAQ,GNAS,GNE,GNPAT,GPD1L,GPD2,GPHN,GPNMB,GPX1,GPX4,GSN,GSTK1,GTF2I,H1F0,HADHA,HAT1,HAVCR2,HCK,HDAC5,HELLS,HEXA,HEXB,HFE,HGS,HIF1A,HIPK2,HK2,HLA-A,HLA-DMA,HLAG,HLTF,HMGCR,HMOX1,HPGD,HRH2,HS6ST1,HSBP1,HSD17B4,HSD17B7,ICA1,ICAM1,IDH1,IDO1,IFIH1,IFIT2,IFNGR1,IFT46,IGF1,IGF1R,IGF2R,Igtp,IKBKE,IL12RB1,IL15,IL15RA,IL18,IL1A,IL1B,IL1RL1,IL1RN,IL4R,IL6,ILK,ING4,INPP5B,INSIG1,INSR,INTU,IRAK2,IRAK3,IRF1,IRF2,IRF5,IRF7,IRF8,Irgm1,ISG15,ITGA6,ITGA9,ITGB1,ITGB3,ITPR1,IVNS1ABP,JAK2,JMJD6,JUP,KAT2B,KAT8,KCNAB1,KCNAB2,KCNJ2,KDM6B,KDR,KEAP1,KIAA0586,KIF1B,KIF3B,KIFAP3,KLF4,KLF6,Klrk1,KMT2C,KMT5C,L1CAM,LAMC1,LAMTOR2,LCP2,LFNG,LHX2,LIFR,LIG1,LIMA1,LMNA,LMNB2,LMO2,LMO4,LPAR1,LPAR6,LRP1,LRP5,Ly6a (includes others),LYN,MADD,MAFB,MAFF,MAFG,MAFK,MAML3,MAN2A2,MAP1LC3B,MAP2K1,MAP3K1,MAP3K12,MAPK14,MAPK3,MAPK7,MAPK9,MARCKSL1,MARCO,MAX,MB21D1,MBNL2,MCM2,MECP2,MED13,MEF2C,MERTK,MIA3,mir-29,MKL2,MMP12,MMP14,Mocs1,MRC1,MSH2,MSH3,MSRA,Mt1,MTDH,MTERF3,MTHFD2,MTHFR,MTM1,MTSS1,MTUS1,MUT,MYC,MYD88,MYL6,NBN,NCAPH,NCF1,NDEL1,NDST1,NEDD4L,NEK7,Nes,NF2,NFAT5,NFE2L1,NFIL3,NFKB1,NFKB2,NFKBIA,NFKBIB,NFKBIZ,NINJ1,NLRP3,NOD2,NOS2,NPAS4,NPM1,NR1H3,NR3C1,NRAS,NRP1,NRP2,NSF,NUMA1,NUP98,OASL,OAT,PAFAH1B1,PAM,PANK1,PANK3,PARP1,PARP3,PATZ1,PCCA,PCYT2,PDCD10,PDCD4,PDE4B,PDGFA,PDGFB,PEA15,PELI1,PFKFB3,PHF21A,PIGT,PIK3CG,PILRA,PILRB,PIP5K1C,PKD2,PLAGL2,PLAU,PLCB3,PLEC,PLK3,PLXND1,PML,PNPLA2,POLD1,PON3,POT1,Pot1b,Pou3f1,PPARG,PPARGC1B,PPIA,PPP1R15A,PPP1R15B,PRKACA,PRKACB,PRKCH,PRKCI,

Continues on next page

Categories	Diseases or Functions Annotation	p-Value	Predicted Activation State	Activation z-score	Molecules
Continued					PRKG1,PRKRA,PRMT5,PRNP,PROCR,PRPF19,PSMB4,PSMC1,PSMC2,PSMC4,PSMD4,PSTPIP1,PTGES,PTGS1,PTGS2,PTK2,PTPN2,PTPRS,QDPR,RAB31,RAC3,RAD51,RAD54L,RALA,RAPGEF2,RASA3,RASSF1,RBBP8,RBL1,RBMS1,RCAN1,RCOR1,RDH10,REL,RELB,REST,RFK,RGPD4 (includesothers),RGS10,RHBDF2,RHOA,RICTOR,RNASEH2B,RNASEL,RNF31,Rpl29 (includes others),RPL4,RPSA,RPTOR,RTN4,RXRA,S100A4,S1PR1,SACM1L,SATB2,SBNO2,SC5D,SCARB1,SCARB2,Scd2,SEL1L,SELENOP,SENP1,SEPT9,SERP1,SERPING1,SERTAD1,SH2D3C,SH3PXD2A,SIGMAR1,SIK3,SIRT6,SIVA1,SKI,SKIL,SLAMF6,SLC11A2,SLC19A1,SLC23A2,SLC25A13,SLC25A37,SLC2A1,SLC2A3,SLC2A8,SLC31A1,SLC35D1,SLC3A2,SLC40A1,SLC7A5,SLIRP,SMG1,SMURF1,SNAPIN,SOCS1,SOCS3,SOD2,SOX7,SP4,SPP1,SPRY2,SQSTM1,SREBF2,SRGAP3,ST7,ST8SIA4,STAB1,STAT1,STAT2,STAT3,STK40,STXBP3,SULF2,SUSD6,SYK,SYNJ1,TANK,TAP1,TBC1D4,TBK1,TCEA1,TCF12,TCF7L2,TDG,TF,TFPI,TGFB1,TGFBR1,TGIF1,TGM2,TGS1,THBD,THBS1,TIMP2,TIPARP,TJP2,TKT,TLR3,TLR9,TMOD1,TNF,TNFAIP3,TNFRSF14,TNFRSF1A,TNFSF10,TNIP1,TOP2A,Tpm1,TPP1,TRAF2,TRAF3,TRAFD1,TREX1,TRIM21,TRIO,TROVE2,TRPM2,TRPS1,TTL,TXN,TYK2,UHRF1,ULK2,UNC13D,USF2,USP18,USP25,VASP,VAV1,VAV3,VCAN,VCL,VEGFA,VKORC1,WDR1,WRN,XPC,ZC3H12A,ZFP36,ZMPSTE24,ZNF274,ZNF281
Carbohydrate Metabolism	metabolism of carbohydrate	3.08E-14	Decreased	-2.019	Abcb1b,ABHD12,ACADM,ACBD6,ADORA2A,ADRB2,AKR1B1,AKT1,AKT2,ALDH2,ALDOC,ALG2,ANXA1,AOAH,ATF4,B3GALNT1,B3GNT3,B3GNT8,B4GALNT1,B4GALT7,B4GAT1,CCL2,CCL5,CD28,CD40,CD44,CDIPT,CEBPA,CEBPB,CEPT1,CERS5,CHST11,CLN6,Cmah,CP,CSF1,CSF1R,CTBP2,CXCL10,CXCL3,DBI,DCXR,DHDH,DPAGT1,DYRK2,EDN1,ENPP1,EPHX1,ETNK1,EXT2,EXTL2,FAS,FCGR2B,FIG4,FOXO1,FPR1,FPR2,GALK2,GALM,GFPT1,Gk,GLO1,Gm21596/Hmgb1,GMDS,GNAQ,GNE,GNPDA1,GUSB,HAGH,HDAC5,HECTD4,HEXA,HEXB,HIF1A,HK2,HLA-A,HS6ST1,ICAM1,IDH1,IGF1,IGF1R,IL18,IL1A,IL1B,IL1RN,IL6,INSR,ITGB1,JAK2,KHK,LAT,LIPE,LPIN1,LRP5,LYN,MAN2B1,MAN2B2,MAPK14,MDP1,MECP2,MMP12,MTM1,MYC,MYD88,NAGA,NAGK,NANS,NCS1,NDST1,NFKB1,NOS2,OAS1,PARP1,PCYT2,PDGFA,PDGFB,PFKFB3,PFKL,PFKP,Pgap2,PGM1,PHKA2,PHKB,PI4K2A,PIGQ,PIGV,PIK3CG,PIM2,PIP5K1C,PITPNM1,PLA2G15,PLAU,PLCB2,PLCB3,PLD1,PLEK,PML,PPARG,PPP1R15A,PRKAG2,PRKG1,PRPS1,PRPS2,PTK2,PYGL,PYURF,RALA,RGS10,RGS2,RHOA,S1PR1,SACM1L,SCAP,SCD,SELENOI,SERP1,SGSH,SH3GLB1,SH3KBP1,SIAE,SIK3,SIRT6,SLC16A3,SLC23A2,SLC2A1,SLC2A8,SLC35D1,SOCS1,SOCS3,SORD,SPP1,SREBF2,ST3GAL2,ST3GAL5,ST6GAL1,ST8SIA4,STAT3,STK40,SULF2,SYK,SYNJ1,TCF7L2,TGFB1,TGFBR1,THBD,TKT,TNF,TNFRSF1A,TNFSF12,XYLB,XYLT1
	synthesis of carbohydrate	1.66E-09	Decreased	-2.355	ACADM,ACBD6,ADORA2A,ADRB2,AKR1B1,AKT1,AKT2,ALG2,ANXA1,ATF4,B3GALNT1,B3GNT3,B3GNT8,B4GALNT1,B4GALT7,B4GAT1,CCL2,CCL5,CD40,CDIPT,CEBPA,CEBPB,CEPT1,CERS5,CHST11,CSF1,CSF1R,CTBP2,CXCL10,CXCL3,DBI,DPAGT1,DYRK2,EDN1,ENPP1,ETNK1,EXT2,EXTL2,FAS,FOXO1,FPR1,FPR2,GFPT1,GMDS,GNAQ,GNE,GNPDA1,HEXA,HLA-A,HS6ST1,ICAM1,IDH1,IGF1,IGF1R,IL18,IL1A,IL1B,IL1RN,IL6,INSR,ITGB1,JAK2,LAT,LIPE,LYN,MAN2B1,MTM1,MYC,MYD88,NANS,NCS1,NDST1,NFKB1,NOS2,PARP1,PCYT2,PDGFA,PDGFB,Pgap2,PI4K2A,PIGQ,PIGV,PIK3CG,PIP5K1C,PITPNM1,PLAU,PLCB2,PLCB3,PLD1,PLEK,PPARG,PPP1R15A,PRKG1,PRPS1,PRPS2,PTK2,PYGL,PYURF,RALA,RGS10,RGS2,RHOA,S1PR1,SCAP,SCD,SELENOI,SH3GLB1,SH3KBP1,SIK3,SIRT6,SLC35D1,SOCS1,SOCS3,SORD,SPP1,SREBF2,STAT3,SYK,TCF7L2,TGFB1,TGFBR1,THBD,TKT,TNF,TNFRSF1A,XYLT1
Infectious Diseases	Bacterial Infections	1.72E-16	Decreased	-3.007	ACOD1,ADCY7,ADH5,ADRB2,AIM2,AKT1,AKT2,ALOX5AP,ANXA1,ARID5A,BAHD1,BCL3,BIRC3,C1QBP,C3,C5AR1,CAPG,CASP1,CASP4,CBY1,CCL2,CCL3L3,CCR2,CCRL2,CD14,CD274,CD28,CD4,CD40,CD44,CD47,CD83,CEBPB,CERK,CHRM3,Cux1,CXCL10,Cxcl11,CXCL16,Cxcl9,CXCR3,CYBB,CYLD,CYP51A1,CYSLTR2,EBI3,FCGR1A,FCGR2B,FCGR3A/FCGR3B,Fcna,FPR1,GCA,Gm21596/Hmgb1,HCK,HIF1A,HLA-DMA,HLA-E,HLA-G,HMGCR,HRH2,ICAM1,IFNGR1,IL12RB1,IL15,IL18,IL1B,IL1RN,IL23R,IL27,IL6,IRAK2,IRF5,IRF7,IRF8,Irgm1,ITGAL,JDP2,MARCO,MMP12,MMP14,MYD88,NCF1,NFKB1,NFKBIB,NLRP3,NOD1,NOD2,NOS2,NR1H3,NR3C1,PARP1,PDE4B,PDE7B,PER1,PILRB,PLA2G7,PLCB2,PML,POLA1,POLD1,PRKCE,PSMB4,PTGS1,PTGS2,RAPH1,RASA4,REL,RELB,RHBDF2,RIPK2,SCARB2,SERPING1,SIGMAR1,SLAMF6,SLAMF8,SLC11A1,SOCS1,SP110,SPP1,ST6GAL1,STAT1,STAT3,TAP1,TGFB1,TGM2,TLR1,Tlr11,TLR6,TLR9,TNF,TNFAIP3,TNFRSF1A,TNFSF10,TNFSF12,TOP2A,TRAFD1,Trim30a/Trim30d,TRPM2,TYK2,USP18,VAV1,ZFP36
