# Supplementary material for: Regulation of mycobacterial infection by macrophage Gch1 and tetrahydrobiopterin
Source: Nat Commun. 2018 Dec 20;9:5409. doi: 10.1038/s41467-018-07714-9 (PMC6302098; doi:10.1038/s41467-018-07714-9)
Supplement: Supplementary file 7 — Supplementary Dataset 4 [file 41467_2018_7714_MOESM7_ESM.pdf]

Cellular functions significantly modulated in infected *Gch1*$^{fl/fl}$Tie2cre macrophages using Ingenuity Pathway Analysis

Categories	Diseases or Functions Annotation	p-Value	Predicted Activation State	Activation z-score	Target molecules in dataset
Cell-To-Cell Signaling	recruitment of myeloid cells	1.29E-11	Increased	2.187	ACVRL1,APOE,ATG7,C5AR1,CAT,CCL3L3,CCL4,CCR7,CD14,CD300LB,CD36,CD47,CSF2,CXCL3,EDN1,FCGR2B,FUT8,GDF15,HAO1,IFNAR1,IL1A,IL1R1,IL2RA,IL6,IL6R,ITGB2,KITLG,LGALS3,LSP1,LY96,NLRP3,NOTCH1,NTN1,OLR1,PIK3R5,RIPK2,SIGLEC9,THBS1,TIRAP,TLR2
	recruitment of granulocytes	1.06E-12	Increased	2.24	ACVRL1,APOE,ATG7,C5AR1,CAT,CCL3L3,CCL4,CCR7,CD14,CD300LB,CD47,CSF2,CXCL3,EDN1,FCGR2B,GDF15,HAO1,IFNAR1,IL1A,IL1R1,IL6,IL6R,ITGB2,KITLG,LGALS3,LSP1,LY96,NLRP3,NTN1,OLR1,PIK3R5,RIPK2,SIGLEC9,THBS1,TIRAP,TLR2
	recruitment of leukocytes	7.02E-12	Increased	2.006	ACVRL1,APOE,ATG7,C5AR1,CAT,CCL3L3,CCL4,CCR7,CD14,CD300LB,CD36,CD47,CSF2,CXCL3,EDN1,F13A1,FCGR2B,FUT8,GDF15,HAO1,HDC,IFNAR1,IL1A,IL1R1,IL2RA,IL6,IL6R,IRF5,ITGA4,ITGB2,KITLG,LGALS3,LSP1,LY96,NLRP3,NOTCH1,NTN1,OLR1,PIK3R5,RIPK2,S1PR2,SIGLEC9,THBS1,TIRAP,TLR2
	recruitment of phagocytes	5.27E-13	Increased	2.209	ACVRL1,APOE,ATG7,C5AR1,CAT,CCL3L3,CCL4,CCR7,CD14,CD300LB,CD36,CD47,CSF2,CXCL3,EDN1,F13A1,FCGR2B,FUT8,HAO1,IFNAR1,IL1A,IL1R1,IL2RA,IL6,IL6R,IRF5,ITGB2,LGALS3,LSP1,LY96,NLRP3,NOTCH1,NTN1,OLR1,PIK3R5,RIPK2,SIGLEC9,THBS1,TIRAP,TLR2
	recruitment of neutrophils	1.05E-12	Increased	2.165	ACVRL1,APOE,ATG7,C5AR1,CAT,CCL3L3,CCL4,CCR7,CD14,CD300LB,CD47,CSF2,CXCL3,EDN1,FCGR2B,HAO1,IFNAR1,IL1A,IL1R1,IL6,IL6R,ITGB2,LGALS3,LSP1,LY96,NLRP3,NTN1,OLR1,PIK3R5,RIPK2,SIGLEC9,TIRAP,TLR2
	binding of leukocyte cell lines	1.80E-04	Increased	2.079	CADM1,CD47,CSF2,ITGA4,ITGB2,MSR1,PLAU,SDC4,TLR2
	activation of neutrophils	2.26E-07	Increased	2.746	C5AR1,CCL4,CD14,CFH,CSF2,CXCL3,EDN1,IL1A,IL6,ITGB2,PF4,SAA1,SIGLEC9,SLC11A1,TLR2
	activation of granulocytes	4.50E-08	Increased	2.522	C5AR1,CCL4,CD14,CD300A,CFH,CSF2,CXCL3,EDN1,FCGR2B,IL1A,IL6,ITGB2,KITLG,PF4,SAA1,SIGLEC9,SLC11A1,TLR2
Inflammatory Response	degranulation of phagocytes	5.39E-06	Increased	2.161	ARAP3,CCL3L3,CCL4,CD300A,CD9,CXCL3,FCGR2B,ITGB2,KITLG,LAT2,Milr1,MTMR4,MYO1F,NR4A3,PAK1,PF4,PLAT,PTGDR,S1PR2,TLR2
	degranulation of cells	3.84E-04	Increased	2.099	ARAP3,C5AR1,CCL3L3,CCL4,CD300A,CD9,CXCL3,FCGR2B,ITGB2,KITLG,LAT2,Milr1,MTMR4,MYO1F,NR4A3,PAK1,PF4,PLAT,PTGDR,S1PR2,TLR2
Cellular Movement	infiltration by myeloid cells	2.90E-07	Increased	2.166	APOE,C5AR1,CCL4,CCR7,CD14,CD36,CD47,CFH,CSF2,CXCL3,DUSP10,EDN1,IL1A,IL1R1,IL2RA,IL6,IL6R,IRF5,ITGB2,KITLG,KNG1,LGALS3,MSR1,NLRP3,NR1H2,NTN1,PF4,PLA2G7,PLAT,PLAU,PLCB3,S1PR2,SAA1,TLR2,TNFSF4
	cellular infiltration by mononuclear leukocytes	2.10E-04	Increased	2.179	APOE,CCL3L3,CCL4,CCR7,CIITA,CSF2,ESR1,IL2RA,IL6,IRF5,ITGA4,MMP14,NLRP3,NR1H2,NTN1,PF4,RIPK3,SAA1,TNFSF4
	cellular infiltration by granulocytes	2.35E-06	Increased	2.132	APOE,C5AR1,CCL4,CCR7,CD14,CD36,CD47,CFH,CSF2,CXCL3,IL1A,IL1R1,IL2RA,IL6,IL6R,ITGB2,KNG1,LGALS3,MSR1,NLRP3,NTN1,PF4,PLA2G7,PLAT,PLCB3,TLR2
	Lymphocyte migration	1.77E-04	Increased	2.073	CCL3L3,CCL4,CCR7,CD151,CD47,CSF2,CXCL3,HDC,IFNAR1,IL2RA,IL6,IRF5,ITGA4,ITGB2,JAK2,KCNN4,NLRP3,NOTCH1,NR1H2,NTN1,PLAU,PLCB3,RAPGEF1,RIPK3,S1PR2,SAA1,SEMA4D,SERP1,THBS1,TLR2,TNFSF4

Continues on next page

Categories	Diseases or Functions Annotation	p-Value	Predicted Activation State	Activation z-score	Target molecules in dataset
Continued					
	Lymphocyte migration	1.77E-04	Increased	2.073	CCL3L3,CCL4,CCR7,CD151,CD47,CSF2,CXCL3,HDC,IFNAR1,IL2RA,IL6,IRF5,ITGA4,ITGB2,JAK2,KCNN4,NLRP3,NOTCH1,NR1H2,NTN1,PLAU,PLCB3,RAPGEF1,RIPK3,S1PR2,SAA1,SEMA4D,SERP1,THBS1,TLR2,TNFSF4
	cell movement of lymphocytes	5.21E-05	Increased	2.069	CCL3L3,CCL4,CCR7,CD151,CD47,CSF2,CXCL3,ESR1,HDC,IFNAR1,IL2RA,IL6,IRF5,ITGA4,ITGB2,JAK2,KCNN4,MMP14,NLRP3,NOTCH1,NR1H2,NTN1,PF4,PLAU,PLCB3,PTGDR,RAPGEF1,RIPK3,S1PR2,SAA1,SAMSN1,SEMA4D,SERP1,THBS1,TLR2,TNFSF4
	cellular infiltration by phagocytes	2.96E-06	Increased	2.199	APOE,C5AR1,CCL4,CD14,CD36,CFH,CSF2,CXCL3,DUSP10,EDN1,IL1A,IL1R1,IL2RA,IL6,IL6R,IRF5,ITGB2,KITLG,LGALS3,NLRP3,NR1H2,NTN1,PF4,PLAT,PLAU,PLCB3,S1PR2,SAA1,TLR2,TNFSF4
	cell movement of neutrophils	6.06E-08	Increased	2.161	APOE,ARAP3,C5AR1,CCL3L3,CCL4,CCR7,CD14,CD151,CD36,CD47,CFH,CSF2,CTTN,CXCL3,EDN1,IL1A,IL1R1,IL2RA,IL6,IL6R,ITGA4,ITGB2,LGALS3,LSP1,MYO1F,NLRP3,NTN1,PF4,PIK3R5,PLAT,PLAU,PLCB3,SAA1,TIRAP,TLR2,TREM3
Endocrine System Disorders, Metabolic Disease	insulin resistance	3.87E-05	Increased	2.699	Akr1b7,Apoc3,APOE,ARFGEF3,ATG7,CA13,CAT,CCR7,CD36,COL5A3,CYP4A11,DUSP10,ESR1,FASN,HMGA1,IFNAR1,IFNAR2,IL1R1,IL6,JAK2,LIPA,mir-34,NLRP3,NOTCH1,PPARGC1A,PRKAB2,SLC6A6,THBS1,TLR2,TP53INP1,TPH2
Hematological Disease	neutrophilia	2.35E-04	Increased	2.07	CXCL3,IL1A,IL1R1,IL6,ITGB2,JAK2,LGALS3,NLRP3,TLR2
Lipid Metabolism	concentration of cholesterol	8.70E-06	Increased	2.618	AEBP1,Akr1b7,APOA5,Apoc3,APOE,CD36,CSF2,CYP27A1,DBI,DHCR24,ESR1,FDFT1,HMGA1,HP,IL6,INSIG1,LDLRAP1,LGALS3,LIPA,MSR1,NR1H2,OLR1,PHYH,PPARGC1A,PTGDR,SAA1,SC5D,SREBF2,STARD4,TLR2,UBIAD1
	quantity of steroid	1.78E-04	Increased	2.498	ABCC4,AEBP1,Akr1b7,APOA5,Apoc3,APOE,CD36,CSF2,CYP27A1,CYP3A7,DBI,DHCR24,EDN1,ESR1,FDFT1,HMGA1,HP,IL1A,IL1R1,IL6,INSIG1,KNG1,LDLRAP1,LGALS3,LIPA,MSR1,NKX2-1,NR1H2,OLR1,PHYH,PLAT,PPARGC1A,PTGDR,SAA1,SC5D,SLC23A2,SREBF2,STARD4,TLR2,UBIAD1
	synthesis of fatty acid	1.93E-05	Increased	2.319	ABCD3,ACSS2,APOA5,APOE,C5AR1,CADM1,CD14,CD36,CSF2,CXCL3,CYP27A1,CYP4A11,EDN1,EDNRB,FADS2,FASN,FCGR2B,IGFBP7,IL1A,IL6,INSIG1,JAK2,KITLG,KNG1,NR1H2,NTN1,OLR1,PPARGC1A,S1PR2,SIGLEC9,TLR2
	synthesis of eicosanoid	1.81E-05	Increased	2.286	APOE,C5AR1,CADM1,CD14,CD36,CSF2,CXCL3,CYP4A11,EDN1,EDNRB,FADS2,FASN,FCGR2B,IGFBP7,IL1A,IL6,JAK2,KITLG,KNG1,NTN1,OLR1,S1PR2,SIGLEC9,TLR2
	exposure of phospholipid	4.04E-05	Increased	2.007	ANXA7,CD47,CD9,CSF2,EDN1,EDNRB,IL1A,PAK1
	synthesis of terpenoid	1.11E-05	Increased	2.054	ABCC4,ALG12,APOE,CSF2,CYP27A1,CYP51A1,DBI,DHCR24,EDN1,ESR1,ETV1,FDFT1,G6PD,GDF15,HDC,IDI1,IGFBP7,IL1A,IL6,INSIG1,JAK2,KITLG,NR1H2,NR4A3,PDSS1,PPARGC1A,SREBF2,STARD4,TLR2
	synthesis of steroid	1.24E-05	Increased	2.054	ABCC4,APOE,CSF2,CYP27A1,CYP51A1,DBI,DHCR24,EDN1,ESR1,ETV1,FDFT1,G6PD,GDF15,HDC,IDI1,IGFBP7,IL1A,IL6,INSIG1,JAK2,KITLG,NR1H2,NR4A3,PPARGC1A,SREBF2,STARD4,TLR2

Categories	Diseases or Functions Annotation	p-Value	Predicted Activation State	Activation z-score	Target molecules in dataset
Continued					
Metabolic Disease	glucose metabolism disorder	3.16E-07	Increased	2.772	9930111J21Rik2,ABCD3,AEBP1,Akr1b7,Apoc3,APOE,ARFGEF3,ATG7,C130026I21Rik (includes others),CA13,CAPSL,CAT,CCL3L3,CCL4,Ccl8,Ccl9,CCR7,CD180,CD300A,CD36,CFH,CHN2,CIITA,COL5A3,CP,CREBBP,CSF2,CTSH,CYP4A11,DUSP10,DYRK1A,ECHS1,EDN1,EDNRB,ESR1,FADS2,FASN,FCGR2B,G6PD,GABRG1,GDF15,GSS,HDC,HLA-DMA,HLA-DMB,HLA-DQA1,HLA-DQB1,HLA-DRB5,HMGA1,HP,IFNAR1,IFNAR2,IGFBP7,IL1A,IL1R1,IL2RA,IL6,IRF1,ITGA4,ITGB2,JAK2,KLF4,LGALS3,LIPA,MAP3K3,MGMT,mir-135,mir-154,mir-27,mir-329,mir-34,MR1,MSR1,MYO1F,NLRP3,NOTCH1,NR1H2,NR4A3,OLR1,PAK1,PCSK1,PDLIM4,PLBD1,PPARGC1A,PPP1R15B,PRKAB2,PSMD6,SETD7,SIRPB1,SKP2,SLC25A13,SLC6A6,SREBF2,STK32C,THBS1,TLR2,TMEM116,TNFSF4,TP53INP1,TPH2,UBR7
Organismal Injury and Abnormalities	cytosis of blood cells	1.35E-04	Increased	2.063	ABL2,CXCL3,Hamp/Hamp2,HDC,IFNAR1,IFNAR2,IL1A,IL1R1,IL6,ITGA4,ITGB2,JAK2,LGALS3,NLRP3,RRM2B,TLR2
Protein Synthesis	quantity of protein in blood	1.56E-06	Increased	2.206	Akr1b7,Apoc3,APOE,ARFGEF3,BBS12,C5AR1,CD14,Cd33,CD36,COL5A3,CSF2,CYP27A1,ESR1,GCLM,GPR68,HDC,HMGA1,IFNAR1,IFNAR2,IL1R1,IL6,IL6R,IRAK2,IRF1,IRF5,ITGB2,LAT2,LDLRAP1,LGALS3,LIPA,NKX2-1,NLRP3,NR1H2,OLR1,PCSK1,PER1,PHYH,PPARGC1A,PRKAB2,PTPRS,SAMSN1,SERP1,SLC25A13,SRXN1,STARD4,TIRAP,TLR2,TP53INP1,TPH2,UBIAD1
Cell Death and Survival	apoptosis of hematopoietic cells	1.23E-04	Decreased	-2.092	BAK1,BBC3,CASP2,CCL3L3,CCL4,CREBBP,CSF2,EPHA4,FCGR2B,IL6,IRF8,KITLG,KLF13,LYL1,MDM2,NOTCH1,PF4,PIM1,PML,RIPK3,SOX4,ST14
	cell death of hematopoietic cells	2.63E-05	Decreased	-2.387	ABCC4,BAK1,BBC3,CASP2,CCL3L3,CCL4,CREBBP,CSF2,EPHA4,FCGR2B,IL6,IRF8,KITLG,KLF13,LGALS3,LYL1,MDM2,MGMT,NOTCH1,PF4,PIM1,PML,RIPK3,SOX4,ST14
	apoptosis of hematopoietic progenitor cells	4.29E-04	Decreased	-2.521	BAK1,BBC3,CASP2,CCL4,CREBBP,CSF2,EPHA4,FCGR2B,IL6,IRF8,KITLG,KLF13,LYL1,MDM2,NOTCH1,PF4,PML,RIPK3,SOX4,ST14
	cell death of hematopoietic progenitor cells	9.45E-05	Decreased	-2.785	ABCC4,BAK1,BBC3,CASP2,CCL4,CREBBP,CSF2,EPHA4,FCGR2B,IL6,IRF8,KITLG,KLF13,LGALS3,LYL1,MDM2,MGMT,NOTCH1,PF4,PML,RIPK3,SOX4,ST14
Humoral Immune Response, Protein Synthesis	quantity of IgG	4.97E-04	Decreased	-2.456	CCR7,CD180,CD36,CIITA,CSF2,ESR1,FCGR2B,GADD45A,GAPT,HLA-DQB1,IFNAR2,IL2RA,IL6,ITGB2,LGALS3,MAN2A1,Rfx5,RIPK2,SAMSN1,TIRAP,TLR2
