# Supplementary material for: Regulation of mycobacterial infection by macrophage Gch1 and tetrahydrobiopterin
Source: Nat Commun. 2018 Dec 20;9:5409. doi: 10.1038/s41467-018-07714-9 (PMC6302098; doi:10.1038/s41467-018-07714-9)
Supplement: Supplementary file 8 — Supplementary Dataset 5 [file 41467_2018_7714_MOESM8_ESM.pdf]

Upstream Regulators significantly modulated in infected *Gch1*$^{fl/fl}$Tie2cre macrophages using Ingenuity Pathway Analysis

Upstream Regulator	Predicted Activation State	Activation z-score	p-value of overlap	Target molecules in dataset
Ige	Activated	2.439	1.02E-03	CCL3L3,CCL4,Ccl9,CSF2,HDC,IL1A,IL6
IL-17f dimer	Activated	2	7.02E-04	CCL4,CSF2,IL1A,IL6
Ccl2	Activated	2.229	2.38E-04	CCL3L3,CCL4,Ccl9,CD14,CXCL3,IL6,KLF4,SDC4
CCL5	Activated	2.542	4.52E-07	ADGRE5,C5AR1,CCL3L3,CCL4,CD163,CXCL3,HDC,HMGA1,IL6,OLR1,SERPINB2,SQLE,VASP
CSF3	Activated	2.013	1.61E-04	CCL3L3,CCR7,CD14,CXCL3,EDN1,GADD45A,HDC,HLA-DQA1,IL6,ITGB2,KITLG,LY96,PIM1,PIWIL1,SLC20A2,TLR2
IL17A	Activated	2.77	1.65E-03	CCL4,Ccl8,CD14,CD163,CSF2,CTTN,CXCL3,ECHS1,FOSL1,GADD45A,IL1A,IL6,mir-154,NRP1,PLAU,TLR2
IL18	Activated	2.177	1.59E-02	CCL3L3,CCL4,CCR7,CSF2,CXCL3,IL1A,IL2RA,IL6,IRF1,SLC2A3
IL1A	Activated	2.36	1.57E-05	ADAMTS4,CCL4,CSF2,CXCL3,FAM89B,GCH1,GNB4,Hamp Hamp2,HDC,IL1A,IL2RA,IL36G,IL6,KITLG,mir-135,MMP14,PDGFA,PLAU,SAA1,TLR2
IL32	Activated	2.409	1.02E-03	BBC3,CCL4,CD36,CXCL3,IL1A,IL6,ITGB2
MIF	Activated	2.623	2.21E-02	CCL3L3,CCL4,CXCL3,FASN,IL2RA,IL6,ITGA4,TPH2
GNAI3	Activated	2.236	1.45E-04	CCL3L3,CCL4,CSF2,CXCL3,IL6
CCR5	Activated	2.029	9.61E-03	CCL3L3,CCL4,CD9,CXCL3,IL6
IL1	Activated	2.172	6.16E-06	ADAMTS4,APOE,CBR3,CCL4,CCR7,CP,CSF2,CXCL3,CYP27A1,EDN1,HP,IL1R1,IL2RA,IL6,IRF1,ITGB2,KITLG,NTF3,PDGFA,PPARGC1A,SAA1,SDC4,SERPINB2,SLC2A3,TLR2
Mek	Activated	2.18	9.59E-03	APOE,CASP2,ESR1,ETV1,FASN,FOSL1,GDF15,IL6,MANSC1,MDM2,MMP14,PIWIL1,PPARGC1A,THBS1
Tnf (family)	Activated	2.012	1.98E-02	BBC3,CCL4,CD163,CXCL3,IL1A,IL1R1,IL6,SAA1,TLR2
TGFB1	Activated	2.622	1.66E-10	ACVRL1,ADAMTS4,AK1,APOE,ASPN,ATG7,B3GALT2,BBC3,C5AR1,CADM1,CAT,CBR3,CCL3L3,CCL4,Ccl8,CCR7,CD14,CD163,CD300A,CD36,Cdkn1c,CENPA,CFH,CIITA,CSF2,CTSH,CTTN,CXCL3,CYP3A7,DNAJB6,EDN1,EDNRB,F13A1,FLNB,FUT8,GABRG1,GADD45A,GALM,GCLC,GDF15,GNB4,GNG2,GPR85,Hamp/Hamp2,HEBP1,HLA-DMA,HLA-DQA1,HLA-DQB1,HLA-DRB5,HMGA1,IDI1,IGFBP7,IL1A,IL1R1,IL2RA,IL6,IL6R,IRAK2,IRF1,ITGA4,ITGB2,JUND,JUP,KITLG,KLF4,KNG1,LGALS3,MAF,MDM2,MGMT,mir-154,mir-27,mir-34,MMP14,MSMO1,MSR1,NKX2-1,NLRP3,NOTCH1,NR4A3,NRP1,OLR1,PDGFA,PDLIM4,PDLIM5,PDXK,PIM1,PLAT,PLAU,PML,PPARGC1A,Prl2c2 (includes others),S1PR2,SDC4,SERP1,SLC12A7,SLC23A2,SLC2A3,SLC4A2,SOX4,THBS1,TLR2,TMEM184B,TXNRD1,VASP,WDFY3,ZFP36L2
IKBKG	Activated	2.091	1.17E-04	AEBP1,BBC3,Ccl8,Ccl9,CP,CSF2,CXCL3,GCH1,IL1A,IL6,PLD3,Prl2c2 (includes others),SERPINB2,TMEM176B
INSR	Activated	2.503	6.33E-06	ACSL5,CCL4,CD36,CYP51A1,ECHS1,EDN1,FDFT1,GADD45A,GCH1,IDI1,IFITM2,IFNAR2,IL6,LAT2,MARCKSL1,MDM2,mir-154,mir-329,mir-34,mir-368,MSMO1,NRF1,OLR1,PIM1,PPARGC1A,Prl2c2 (includes others),RNF4,RTN2,SC5D,SQLE,SREBF2,Srsf5
RIPK2	Activated	2.262	3.04E-03	CD36,CXCL3,FASN,GPR84,IL6,MARCKSL1,PPARGC1A,SLC2A6
TBK1	Activated	2.813	2.76E-03	CCL3L3,CCL4,CXCL3,EDN1,HDC,IL1A,IL6,TLR2
CYR61	Activated	2.391	3.26E-02	CCL3L3,CCL4,CXCL3,ESR1,IL1A,IL6,PLAU
KRT17	Activated	2	1.82E-02	CCL3L3,CCL4,CXCL3,IL6
MBTD1	Activated	2	3.82E-02	CCL4,CXCL3,SOX4,TLCD2
MYD88	Activated	2.034	1.10E-04	CCL3L3,CCL4,CD14,CSF2,CXCL3,EDNRB,GDF15,HDC,HP,IL1A,IL6,IRF1,IRF8,ITGA4,MMP14,MSR1,OASL,OLR1,SAA1,TLR2
NOD2	Activated	2.403	3.16E-02	CXCL3,GPR84,IL1A,IL6,MARCKSL1,SLC2A6
OSCAR	Activated	2	1.82E-02	CXCL3,IL1A,IL1R1,JAK2
SCAP	Activated	3.066	2.54E-08	ACSL5,ACSS2,CYP51A1,FADS2,FASN,FDFT1,IDI1,INSIG1,MSMO1,SC5D,SQLE,SREBF2,STARD4
TAC1	Activated	2.546	2.52E-04	CCL4,CSF2,CXCL3,HDC,IL1A,IL6,KITLG,MAFB,PLAT,SERPINB2
C3	Activated	2.198	2.38E-02	CCL4,Ccl9,CXCL3,IL1A,IL6
CASP1	Activated	2.207	9.54E-04	CSF2,FASN,IL1A,IL6,RIPK2,SREBF2
CEBPB	Activated	3.228	1.41E-03	AKR1B10,Akr1b7,CCL3L3,CCL4,CD14,CP,CXCL3,DHX9,GADD45A,Hamp/Hamp2,HDC,HIST4H4,HMGA1,HP,IL1A,IL6,MSR1,NRP1,PPARGC1A,RAD18,SERP1,SERPINB2,TMEM176A,TMEM176B,TOP1
FOXL2	Activated	2.429	1.77E-02	CCL3L3,CXCL3,NR4A3,PPARGC1A,SERPINB2,SOX4
GATA2	Activated	2.832	1.01E-03	ADGRE5,AK1,CCL3L3,Cd33,CD36,CSTA,EDN1,F13A1,FCHO1,HDC,IGFBP7,IKZF1,IL6,LYL1,MAFB,MCTP1,MYO18B,Prl2c2 (includes others),SERPINB2,Serpinb6b,SKP2,THBS1,TNFSF4,TREM3,TSPAN32,ZMAT3
HIF1A	Activated	2.244	3.80E-03	APOE,CCR7,Cdkn1c,CXCL3,EDN1,Hamp/Hamp2,HP,IL1A,IL6,ITGB2,JUP,MGMT,NOTCH1,NR4A3,NRF1,NUCKS1,PDGFA,SDC4,SLC2A3,SLC40A1,ST3GAL1,THBS1,TLR2,VASP

Continues on next page

Upstream Regulator	Predicted Activation State	Activation z-score	p-value of overlap	Target molecules in dataset
Continued				
HMGB1	Activated	2.769	8.33E-04	CCL4,CXCL3,FASN,IL1A,IL1R1,IL6,MDM2,TLR2
SIRT2	Activated	2	1.77E-03	FDFT1,IDI1,SC5D,SQLE
SREBF1	Activated	2.02	8.58E-05	ACSL5,ACSS2,APOA5,Bhlhe41,CYP51A1,DBI,FADS2,FASN,FDFT1,G6PD,IDI1,IL6,INSIG1,LGALS3,MSMO1,SC5D,SORBS3,SQLE,STARD4
SREBF2	Activated	2.159	2.76E-08	ACSS2,CYP51A1,DBI,FADS2,FASN,FDFT1,G6PD,IDI1,INSIG1,LIPA,MSMO1,SC5D,SQLE,SREBF2,STARD4
TFAP4	Activated	2	4.95E-02	DYRK1A,ETV1,GDF15,MDM2
ICAM1	Activated	2.442	4.72E-05	CCL3L3,CCL4,CXCL3,IL1A,IL2RA,IL6,ITGA4,NOTCH1
ICOS	Activated	2.183	5.79E-03	CSF2,IL1A,IL2RA,IL6,MAF
NCR2	Activated	2	2.29E-03	CCL4,CSF2,NR4A3,THBS1
TLR2	Activated	2.797	3.19E-03	CCL3L3,CR1L,CSF2,CXCL3,HLA-DQA1,HLA-DRB5,IL2RA,IL6,IRF1,ITGA4,SLC40A1,TLR2
TLR4	Activated	2.606	3.08E-06	BBX,CCL3L3,CCL4,Ccl8,CCR7,CD14,CD163,CSF2,CXCL3,EDN1,FCGR2B,HDC,IL1A,IL2RA,IL6,IRF1,IRF8,MT-ND6,NOTCH1,NR4A3,OASL,PLAT,PML,RIPK2,SLC6A12,SMPDL3A,ST3GAL1,TLR2,TREM3
TLR5	Activated	2.172	5.03E-03	CCL4,CSF2,CXCL3,IL1A,IL6
TLR9	Activated	2.344	1.70E-03	9830107B12Rik/A530064D06Rik,ADAMTS4,CCL3L3,CCL4,CCR7,CD300A,CSF2,CXCL3,EDN1,GCH1,Hamp/Hamp2,IL1A,IL6,IRF1,SMPDL3A
EBI3	Inhibited	-2.425	3.15E-03	CIITA,HLA-DMA,HLA-DMB,HLA-DQA1,IRF1,PIM1
IFNA2	Inhibited	-2.075	3.50E-02	GOT1,IFITM2,IFNAR2,IL2RA,IL6,IRF1,IRF5,MDM2,PLAT,PML,SAMSN1,SERPINB9
IFNB1	Inhibited	-2.362	1.19E-04	BAK1,CCL4,CD14,GNB4,Hamp/Hamp2,IDI1,IFNAR1,IL1R1,IL6,IRF1,NOTCH1,PML,RIPK2,SERPINB2,SIRPB1,SQLE,SREBF2,STARD4,THBS1,Tlr13,TNFSF4
CYP51A1	Inhibited	-2.236	6.58E-05	DHCR24,MSMO1,SC5D,SQLE,SREBF2
FDFT1	Inhibited	-2	3.64E-03	CYP27A1,FASN,SQLE,SREBF2
EPHB6	Inhibited	-2	3.82E-02	CSF2,IL6,mir-27,mir-34
NR1H4	Inhibited	-2.191	2.47E-02	ABCC4,Akr1b7,APOA5,EDN1,FASN,GCLM,IL6,KNG1,NDRG2,PPARGC1A
CORT	Inhibited	-2.412	6.65E-04	CCL3L3,CCL4,CCR7,CXCL3,IL1A,IL6
BCL6	Inhibited	-3.223	2.23E-02	CCL3L3,CCL4,CCR7,CD151,CIITA,CXCL3,GADD45A,IL1A,IL2RA,IL6,IL6R,IRF8,MAF
FOSL1	Inhibited	-2.236	8.33E-04	CCL3L3,CCL4,Ccl8,FOSL1,GCLC,IL6,PLAU,SERPINB2
NFE2L2	Inhibited	-3.139	9.92E-03	ABCC4,ACSL5,AKR1A1,AKR1B10,ARHGEF3,CAT,CD36,CREG1,CXCL3,G6PD,GCLC,GCLM,GSS,GSTM5,HTATIP2,IL1A,IL6,MSMO1,PGD,PPARGC1A,SAA1,SRXN1,TXNRD1
TP73	Inhibited	-3.047	1.13E-03	ATG7,BAK1,BBC3,CADM1,CASP2,Ccl9,Cdkn1c,CXCL3,FASN,G6PD,GADD45A,GPR137B,IL6,IRF5,MDM2,MGMT,MMP14,PML,Prm1,RRM2B,Serpinb6b,THBS1,ZMAT3
WT1	Inhibited	-2.455	1.67E-05	ADGRE5,AMHR2,BAK1,DYRK1A,ESR1,FDFT1,IDI1,IRF8,JUND,JUP,KLF4,LGALS3,MDM2,MMP14,NKX2-1,PDGFA,PIR,SLC2A3,SLC6A6,SQLE,SREBF2,THBS1
RPSA	Inhibited	-2.186	3.74E-03	CSF2,CXCL3,IL1A,IL1R1,IL6
B2M	Inhibited	-2.646	6.36E-07	CCL3L3,CSF2,Hamp/Hamp2,HLA-DQA1,HLA-DQB1,HLA-DRB5,IL6,TAPBP
SIGIRR	Inhibited	-2	7.02E-04	CCL3L3,IL1A,IL1R1,IL6
ABCA1	Inhibited	-2.572	8.53E-05	ABCA8,APOE,CCL3L3,CXCL3,CYP51A1,FDFT1,IL6,SREBF2
APOE	Inhibited	-2.621	4.11E-03	APOE,C5AR1,CAT,CCL3L3,CCL4,CD36,CXCL3,EDN1,FCGR2B,IL1A,IL6,LIPA,MSR1,PLAU,SDC4,SREBF2
UCP1	Inhibited	-2.111	1.04E-02	ATG7,CHCHD10,EMB,FASN,G6PD,GADD45A,GDF15,GOT1,NRF1,PGD,SND1
