# Supplementary material for: Regulation of mycobacterial infection by macrophage Gch1 and tetrahydrobiopterin
Source: Nat Commun. 2018 Dec 20;9:5409. doi: 10.1038/s41467-018-07714-9 (PMC6302098; doi:10.1038/s41467-018-07714-9)
Supplement: Supplementary file 10 — Reporting Summary [file 41467_2018_7714_MOESM10_ESM.pdf]

nature research

Corresponding author(s): Dr Eileen McNeill & Prof Keith M. Channon

Reporting Summary

Nature Research wishes to improve the reproducibility of the work that we publish. This form provides structure for consistency and transparency in reporting. For further information on Nature Research policies, see Authors & Referees and the Editorial Policy Checklist.

Statistical parameters

When statistical analyses are reported, confirm that the following items are present in the relevant location (e.g. figure legend, table legend, main text, or Methods section).

n/a	Confirmed	
☐	☒	The exact sample size (n) for each experimental group/condition, given as a discrete number and unit of measurement
☐	☒	An indication of whether measurements were taken from distinct samples or whether the same sample was measured repeatedly
☐	☒	The statistical test(s) used AND whether they are one- or two-sided<br>*Only common tests should be described solely by name; describe more complex techniques in the Methods section.*
☒	☐	A description of all covariates tested
☐	☒	A description of any assumptions or corrections, such as tests of normality and adjustment for multiple comparisons
☐	☒	A full description of the statistics including central tendency (e.g. means) or other basic estimates (e.g. regression coefficient) AND variation (e.g. standard deviation) or associated estimates of uncertainty (e.g. confidence intervals)
☐	☒	For null hypothesis testing, the test statistic (e.g. F, t, r) with confidence intervals, effect sizes, degrees of freedom and P value noted<br>*Give P values as exact values whenever suitable.*
☒	☐	For Bayesian analysis, information on the choice of priors and Markov chain Monte Carlo settings
☒	☐	For hierarchical and complex designs, identification of the appropriate level for tests and full reporting of outcomes
☐	☒	Estimates of effect sizes (e.g. Cohen's d, Pearson's r), indicating how they were calculated
☐	☒	Clearly defined error bars<br>*State explicitly what error bars represent (e.g. SD, SE, CI)*

Our web collection on statistics for biologists may be useful.

Software and code

Policy information about availability of computer code

Data collection	Flow Cytometry: Summit 4.3; Gene Array: Affymetrix Genechip Command Console 4.0 for GeneTitan.
Data analysis	Flow Cytometry: Flow Jo version 6 (peritonitis and cell counts) and 10 (supplementary data); Statistics: Prism 6; Microarray analysis: Limma, Bioconductor (R version 2.14), Ingenuity Pathway Analysis (version 01-08), GenePattern (v3.9.10), Cytoscape (v.3.5.1)

For manuscripts utilizing custom algorithms or software that are central to the research but not yet described in published literature, software must be made available to editors/reviewers upon request. We strongly encourage code deposition in a community repository (e.g. GitHub). See the Nature Research guidelines for submitting code & software for further information.

Data

Policy information about availability of data

All manuscripts must include a data availability statement. This statement should provide the following information, where applicable:

- Accession codes, unique identifiers, or web links for publicly available datasets
- A list of figures that have associated raw data
- A description of any restrictions on data availability

For the human studies, raw and normalized expression data have been deposited at Gene Expression Omnibus under the accession number GSE98550. For the mouse studies, raw and normalized expression data have been deposited at GEO under the accession number GSE107543. Source data for western blot and DNA

gels and flow cytometry gating in Figures 1 and 2 is available in the supplementary methods file. Full lists of genes and genesets passing significance thresholds are provided as supplementary datasets.

Field-specific reporting

Please select the best fit for your research. If you are not sure, read the appropriate sections before making your selection.

☒ Life sciences ☐ Behavioural & social sciences ☐ Ecological, evolutionary & environmental sciences

For a reference copy of the document with all sections, see nature.com/authors/policies/ReportingSummary-flat.pdf

Life sciences study design

All studies must disclose on these points even when the disclosure is negative.

Sample size	M.tb aerosol challenge: For M.tb. challenge studies 8 mice per group were used. Experiments were designed to have 75% power to detect differences between groups, based on a p value of 0.05. For other in vivo experiment n=5-8 was used based on previous experiments with naive control groups and pilot data to identify the optimal timepoint (peritonitis).
Data exclusions	CFU studies were analyzed by Mann Whitney test, with statistical outliers identified using Grubb's test and removed from the dataset. In this case a single value was removed from the spleen CFU values in Figure 2. For Peritonitis studies any lavage fluid samples showing blood contamination were excluded. All animals were regenotyped after the experiment was completed and for in vitro assays any Gchfl/flTie2cre cell preparations showing incomplete knockout in this conditional system were excluded from analysis.
Replication	The core 6 week M.tb phenotype was replicated in two independent statistically powered experiments. All animals were re-genotyped post experiment either by PCR or by functional assay (Griess Assay for in vitro studies) for every in vivo and primary cell assay. For Gene Array analysis replicate pellets were assayed to confirm cell genotype prior to gene expression assay. Indicator genes showing differences by genotype in the murine gene array were assayed by qPCR in an independent cohort of mice to confirm the finding (Supp Fig 9) and identified pathways and cell functions were assayed to validate findings (Supp Fig 10 and 13) in an independent.
Randomization	Animals are assigned a sequential animal number at weaning prior to genotyping. For in vivo experiments adult female mice >10weeks of age were used for all studies, whole litters were assigned to experimental groups, with heterozygous breeding strategies providing a mix of genotypes within each litter. All in vivo and In vitro samples were assayed and analysed by animal number, rather than my genotype groups.
Blinding	Experiments using littermate animals were performed blinded to the genotypes of the individual animals with samples identified by animal number throughout stimulation and assay and unblinded for statistical analysis. For homozygous strains in vivo experiments scientists were blinded to the genotype by labeling of the cages by a group code, rather than genotype.

Reporting for specific materials, systems and methods

Materials & experimental systems

n/a	Involved in the study
☒	☐ Unique biological materials
☐	☒ Antibodies
☒	☐ Eukaryotic cell lines
☒	☐ Palaeontology
☐	☒ Animals and other organisms
☐	☒ Human research participants

Methods

n/a	Involved in the study
☒	☐ ChIP-seq
☐	☒ Flow cytometry
☒	☐ MRI-based neuroimaging

Antibodies

Antibodies used	All supplier and catalogue details for commercial antibodies are provided in the methods section along with concentrations used in this study.
Validation	Western blot antibodies GTPCH and iNOS were validated against knockout tissues as included in the figures within this manuscript. Flow cytometry antibodies were selected based on the validation data provided by the manufacturer. For immunohistochemistry the anti-iNOS antibody was validated against iNOS-/- samples and this data is included in the figure.

Animals and other organisms

Policy information about studies involving animals; ARRIVE guidelines recommended for reporting animal research

Laboratory animals	Adult mice >10weeks of age were used for all experiments. Infection studies used age-matched cohorts typically 10-14weeks of age at initiation of study. Nos2-/- (C57bl6/J) were obtained from Jackson Labs, C57bl6/J mice were obtained from Charles River, Gchfl/fl and Gchfl/fl Tie2cre mice were produced as littermates in house. Gchfl/fl Tie2cre mice are backcrossed to C57bl/6J for at least 8 generations. Further details of breeding strategy in methods section.
Wild animals	n/a
Field-collected samples	n/a

Human research participants

Policy information about studies involving human research participants

Population characteristics	This study presents additional analysis of a previously reported dataset. The correlates of risk samples used in this study formed part of larger cohort from a phase IIb efficacy trial (ClinicalTrials.gov number NCT00953927) . Publication: Tameris MD, et al. Safety and efficacy of MVA85A, a new tuberculosis vaccine, in infants previously vaccinated with BCG: a randomised, placebo-controlled phase 2b trial. Lancet 381, 1021-1028 (2013). Further details are given in the Methods section of this paper.
Recruitment	This study presents additional analysis of a previously reported dataset. The correlates of risk samples used in this study formed part of larger cohort from a phase IIb efficacy trial (ClinicalTrials.gov number NCT00953927). Publication: Tameris MD, et al. Safety and efficacy of MVA85A, a new tuberculosis vaccine, in infants previously vaccinated with BCG: a randomised, placebo-controlled phase 2b trial. Lancet 381, 1021-1028 (2013).

Flow Cytometry

Plots

Confirm that:

☒ The axis labels state the marker and fluorochrome used (e.g. CD4-FITC).

☒ The axis scales are clearly visible. Include numbers along axes only for bottom left plot of group (a 'group' is an analysis of identical markers).

☒ All plots are contour plots with outliers or pseudocolor plots.

☒ A numerical value for number of cells or percentage (with statistics) is provided.

Methodology

Sample preparation	For peritonitis cells are collected as a single cell solution, for in vitro BMDM experiments cells were cultured on bacteriological plasticware and detatched with ice cold PBS/EDTA5mM solution.
Instrument	Data was collected on a DAKO CYAN ADP analyser
Software	Data was aquired using Summit v4.3 and analysed using Flow Jo version 6 or 10.
Cell population abundance	Cell sorting was not performed.
Gating strategy	The cell population was identified using a standard FSC/SSC setting for primary murine leukocyte analysis. The FSC/SSC gate was validated in pilot studies using CD45 to identify the leukocyte population vs any contaminating debris or red blood cells. Specific cell populations were identified by comparison to isotype control antibodies conjugated with the same fluorochrome. The sequential gating strategy is shown in a supplementary figure.

☒ Tick this box to confirm that a figure exemplifying the gating strategy is provided in the Supplementary Information.
